# Diversity and characterization of culturable fungi associated with the marine sea cucumber *Holothuria scabra*

**DOI:** 10.1371/journal.pone.0296499

**Published:** 2024-01-02

**Authors:** Lakkhana Kanhayuwa Wingfield, Jirawalan Atcharawiriyakul, Ninadia Jitprasitporn

**Affiliations:** Division of Biological Science, Faculty of Science, Prince of Songkla University, Hat Yai, Songkhla, Thailand; Leibniz-Institut fur Naturstoff-Forschung und Infektionsbiologie eV Hans-Knoll-Institut, GERMANY

## Abstract

Fungi associated with the marine echinoderm, *Holothuria scabra*, produces extracellular enzymes and bioactive metabolites, and mycoviruses that could be used for biotechnological and pharmaceutical applications. The species identification based on molecular and morphological characteristics classified the culturable fungi into twenty-three genera belonging to eight orders, Chaetothyriales, Eurotiales, Hypocreales, Mucorales, Mycosphaerellales, Onygenales, Pleosporales and Venturiales, from four classes, Eurotiomycetes, Dothideomycetes, Mucoromycetes and Sordariomycetes of the two phyla Ascomycota and Mucoromycota. The most frequent genera were *Aspergillus* (relative frequency, 45.30%) and *Penicillium* (relative frequency, 22.68%). The Menhinick species richness and Shannon species diversity indices were 1.64 and 2.36, respectively, indicating a high diversity of fungi. An enzymatic production test revealed that sixteen isolates could produce proteases and amylases at different levels. The presence of mycoviruses was detected in eight isolates with different genomic profiles. Thirty-two of the 55 isolates produced antimicrobial metabolites which had an inhibitory effect on various microbial pathogens. Most of these active isolates were identified as *Aspergillus*, *Penicillium* and *Trichoderma*. Notably, *Aspergillus terreus* F10M7, *Trichoderma harzianum* F31M4 and *T*. *harzianum* F31M5 showed the most potent activity against both Gram-positive and Gram-negative bacteria and human pathogenic fungi. Our study represents the first report of the mycobiota associated with the marine echinoderm *Holothuria scabra*.

## Introduction

The sea cucumber *Holothuria scabra*, or sandfish, is found in the seabeds of many tropical waters including the Gulf of Thailand and Andaman Sea. This prized species is widely consumed in China, Japan, Korea, Hong Kong, and Taiwan [1]. The biomolecules produced in its body wall include saponins, and some of the biomolecules found are thought to exhibit several health-related benefits, including anticoagulant, anti-inflammatory and wound healing activities [2]. Bioactive compounds from *H*. *scabra* have demonstrated strong antibacterial and antifungal activities against *Escherichia coli*, *Pseudomonas aeruginosa*, *Staphylococcus aureus*, *Aspergillus niger* and *Candida albicans* [3].

Fungi play an important role in the marine ecosystem and inhabit most marine habitats [4]. Twenty-nine genera of fungi belonging to 24 families in the phylum Ascomycota have been cultured from five genera of sea cucumbers: *Holothuria*, *Cucumaria*, *Stichopus*, *Apostichopus*, and *Eupentacta*. The dominant fungal genus was found to be *Aspergillus*, followed by *Penicillium* (reviewed by [5]). Fungal viruses, or mycoviruses, have been widely detected in all the major fungal phyla, and usually cause asymptomatic infections. However, marine fungi harboring mycoviruses remain poorly investigated despite the increasing awareness of marine fungal diversity. A few studies have presented investigations of mycoviruses associated with the seagrass *Posidonia oceanica* [6] and *Holothuria poli* [7, 8]. In general, mycoviruses do not cause symptoms in their hosts, but some affect mycelial growth, pigmentation, sporulation, pathogenicity and metabolite production [9, 10].

Besides their ecological value, marine-derived fungi have shown potential as source of compounds with pharmaceutical [11–14], cosmeceutical, and nutraceutical properties [15]. Research into new drugs from marine sources often focuses on bioactive molecules produced by marine macro-organisms without considering substrate microbial colonization. However, microorganisms, rather than the animal or plant host, can produce useful metabolites. For instance, a chemotherapeutic Ecteinascidin was found to be produced by a bacterial endosymbiont of the marine tunicate *Ecteinascidia turbinate* [16]. The wide range of properties exhibited by marine-derived fungi includes antibacterial, antifungal, antiviral, anticancer, anti-inflammatory, pro-osteogenic and cytotoxic activities [11–14]; many of which can be attributed to specific enzymes [17]. Investigations of enzymes obtained from marine-derived fungi have identified amylases, glucosidases, laccases, lignin peroxidases, lipases, proteases and xylanases [18–23]. To date, 145 natural products have been isolated from microorganisms associated with sea cucumbers. These compounds include alkaloids, polyketides and terpenoids (reviewed by [5]). Exploiting microorganisms as metabolite and enzyme producers is very advantageous because a fungus can be cultivated *in vitro*, allowing further studies to be conducted without environmental impact. Moreover, the development of fermentation processes for fungal cultivation allows the industrial production and extraction of metabolites [24].

Investigations of the mycobiota present in marine environments of Thailand have mainly focused on sponges, corals, algae, seagrasses, mangrove trees and marine salterns [25–29], but the associated mycobiomes have never been deeply analyzed especially in the echinoderms. In the case of fungi associated with *H*. *scabra*, we still poorly understand their diversity, ecological role and potential to produce secondary metabolites. In this study, we describe the culturable fungal community living in association with *H*. *scabra*. Isolates with biotechnological potential were investigated for their ability to synthetize metabolites for pharmaceutical applications, and enzymes for industrial applications. Studies of mycoviral infections are also significant to the understanding of fungus-virus-host ecological complexes.

## Materials and methods

### *Holothuria scabra* collection

**Fig 1:**
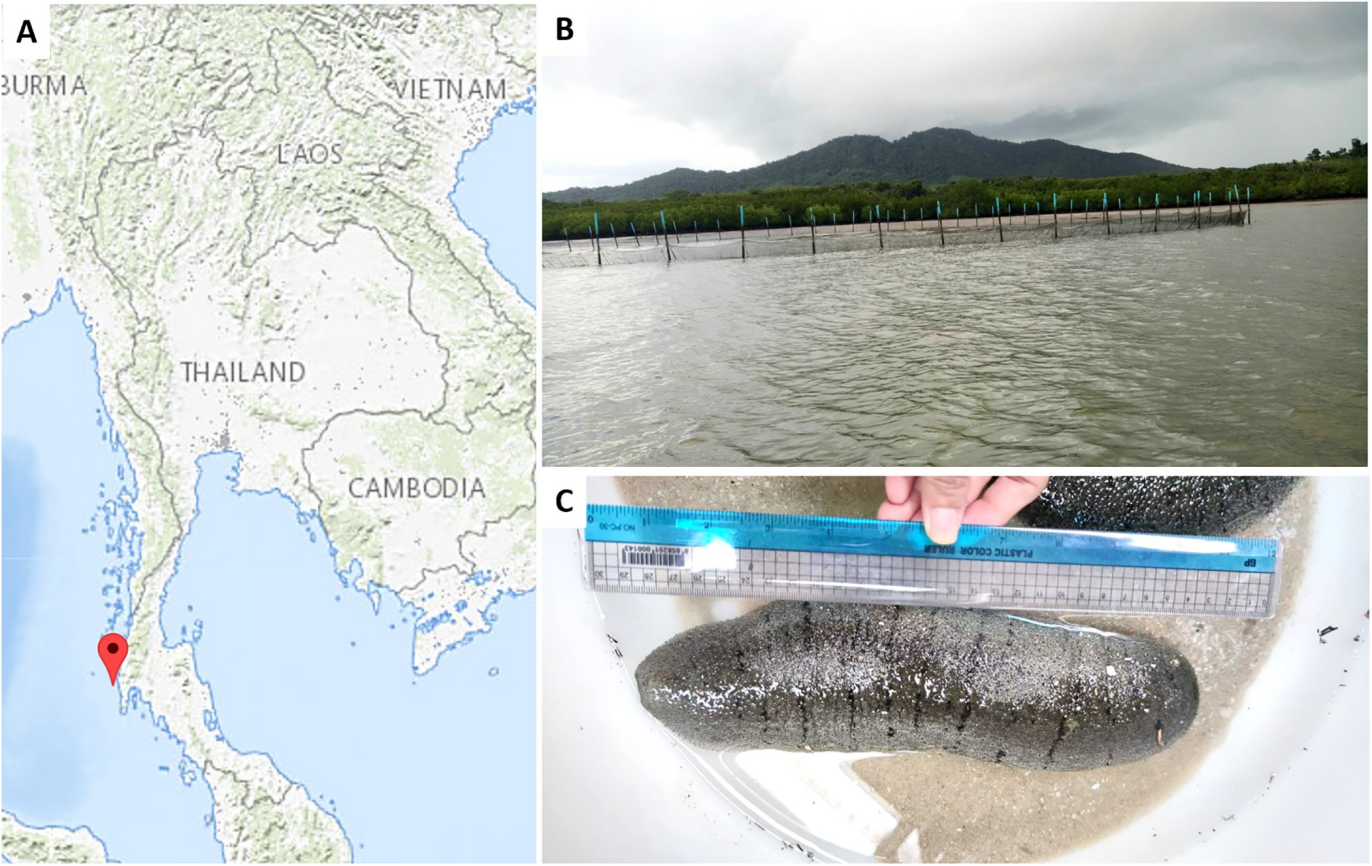
Sampling sites. A) The locations of sampling sites (USGS National Map Viewer; http://viewer.nationalmap.gov/viewer/). B) Characteristics of the sampling sites and neighboring locations in Phang Nga Province, Thailand. C) Physical characteristics of *H*. *scabra*.

Six individuals of *H*. *scabra* were collected in October 2022 from sea cucumber cage cultures along the Andaman Sea coastline of Phang Nga Province in southern Thailand (Fig 1). The average pH of the sea-water at the sites was 6.73. Samples were maintained at 4°C during transportation. Fungal colonization was evaluated on different sea cucumber sections. Before evaluation, specimens were rinsed in sterile seawater, surface sterilized with 70% ethanol, and then subjected to surgical manipulation in a sterile condition to separate the body wall, intestine and faeces. This research project has been approved by Institutional Animal Care and Use Committee, Prince of Songkla University (Ref.AI088/2022).

### Isolation of fungi from *H*. *scabra*

Each sample was homogenized (TissueRuptor II, QIAGEN) and then suspended 1: 10 w/v in phosphate buffer. An aliquot of each sample was dried and the number of colony forming units per gram of dry weight (CFU per gdw) was calculated. One ml of suspension was plated on Corn Meal Agar (CMA) Sea Water (17 g corn meal agar dissolved in 1 L of sterilized artificial seawater, 2% w/v sea salts in ddH_2_O) supplemented with chloramphenicol at 100 μg/ml. Three replicates per sample were performed. Plates were incubated at 24°C in the dark. Fungal colonies were observed periodically for morphological characterization. Strains from each fungal morphotype and from each section were preserved at the Mycology Laboratory, Department of Microbiology, Prince of Songkla University.

### Identification of associated fungal isolates

The identification of isolated fungi was carried out using morphological and molecular approaches. Fungi were initially morphologically identified to the genus and species levels based on their macroscopic and microscopic characteristics described in the specific identification keys of Samson et al. [30], Dugan [31] and Ellis et al. [32]. Molecular identification was performed based on ITS regions of rRNA genes. Genomic DNA was extracted using a protocol described by Wingfield and Atcharawiriyakul [33] and the DNeasy^®^ Plant Mini Kit (QIAGEN, UK), following the protocol provided by the manufacturer.

PCR amplification of fungal ITS regions was carried out using the ITS primer set, ITS5/ITS4N, which amplified a 600–800 bp section of the ITS and had the following sequences: ITS5 (5’-GGAAGTAAAAGTCGTAACAAGG-3’)/ITS4N (5’-TCCTCCGCTTATTGATATGC-3’), for nuclear large subunit rDNA (LSU): LR0R (5′-ACCCGCTGAACTTAAGC-3′)/LR5 (5′-TCCTGAGGGAAACTTCG-3′), and for β-tubulin Bt2a (5′-GGTAACCAAATCGGTGCTGCTTTC-3′)/Bt2b (5′-ACCCTCAGTGTAGTGACCCTTGGC-3′) [34]. Each 50 μl reaction mixture contained 2.5 mM 10xbuffer, 2.0 mM (10 μmol/L) dNTPs, 2.0 mM (5 μmol/L) of each primer, 1.0 mM (3U) Taq DNA polymerase and 1 mM genomic DNA. The PCR reaction was performed using a DNA Engine DYAD ALD 1244 thermocycler (MJ Research, Inc.). PCR amplification condition for ITS was under the following temperature cycling parameters: initial denaturation at 94°C for 5 min; 35 cycles of denaturation at 94°C for 1 min, annealing at 55°C for 1 min and extension at 72°C for 2 min; followed by a final extension at 72°C for 10 min. PCR amplification condition for LSU was under the following temperature cycling parameters: initial denaturation at 95°C for 4 min; 30 cycles of denaturation at 95°C for 30 sec, annealing at 55°C for 30 sec and extension at 72°C for 30 sec; followed by a final extension at 72°C for 5 min. For β-tubulin, the conditions were as follows: 95°C for 5 min, followed by 30 cycles of 95°C for 35 s, 55°C for 50 s, and 72°C for 2 min; an elongation step was performed at 72°C for 7 min. The PCR products were visualized by electrophoresis on a 1% agarose gel in 1XTAE buffer at 100 V for 30 min, purified using the MinElute^®^ Gel Extraction Kit (QIAGEN). DNA sequencing was performed using Sanger method with 3730xl DNA Analyzer (Life technology, Carlsbad, CA, USA) by Macrogen (Seoul, Korea).

The closest matched sequences in the National Centre for Biotechnology Information (NCBI) GenBank database were searched using the BLASTn search tool. To confirm the identity of the isolates, phylogenetic and molecular evolutionary analyses were conducted using MEGA version 11 [35]. Multiple sequence alignments were performed with MUSCLE, and when necessary, sequences were manually edited to maximize the alignment. The phylogenetic tree was inferred using the maximum-likelihood algorithm. The stability of relationships was evaluated by bootstrap analysis with 1,000 replications. Newly generated sequences were deposited in the GenBank database (S1 Table).

### Enzyme assays

Amylase activity was tested on nutrient agar containing 2 g/L of soluble starch. Cultures were incubated for 2 to 5 days, and then flooded with an iodine solution. The presence of amylase was evaluated from the clear zone around the colony [36]. Cellulase activity was tested on a solid medium (7.0 g/L KH_2_PO_4_, 2.0 g/L K_2_HPO_4_, 0.1 g/L MgSO_4_.7H_2_O, 1.0 g/L (NH_4_)_2_SO_4_, 0.6 g/L yeast extract, 10 g/L microcrystalline cellulose and 15 g/L agar) containing 1% cellulose [37]. After incubation, the cultures were further incubated at 50°C for 16 h to accelerate enzyme activity. The cultures were then flooded with 5 mL of 1% Congo red solution and rinsed with distilled water to detect a hydrolysis zone. Protease activity was tested on casein agar medium containing 30% skim milk and 2% agar. Degradation of casein was indicated by a clear zone around the colony [38]. Lipase activity was tested on a solid medium containing Tween 80 (10 g/L peptone, 5 g/L NaCl, 0.1 g/L CaCl_2_.2H_2_O, 17 g/L agar and 10 mL/L Tween 80). Tween 80 was sterilized before addition to the sterile medium. Cultures were kept at 4°C for 12 h after incubation to observe opaque precipitation around the colony [36]. Each enzyme activity was graded with an enzymatic index (EI) where EI = R/r (R being the diameter of the clear zone, and r the diameter of the colony).

### Screening of mycoviruses

Mycovirus dsRNAs were extracted using LiCl fractionation as described by Hull and Covey [39]. dsRNA samples were purified by phenol-chloroform extraction. DNA and ssRNA contamination were removed by sequential DNase I and SI nuclease treatment. The viral genome was proved to be dsRNA by digestion with RNase III. The presence of the dsRNA was checked by electrophoresis in a 1% (w/v) agarose gel in 1X TAE buffer.

### Antimicrobial assays

Fungal isolates were cultivated in Potato Dextrose Broth (PDB) for 21 days at 28°C. The culture broths were used to test antimicrobial activity by the agar well diffusion method [40] against seven pathogenic bacteria (*Micrococcus luteus* (ATCC9341), *Staphylococcus aureus* (ATCC25923), methicillin-resistant *S*. *aureus* (MRSA), *Escherichia coli* (ATCC25922), *Pseudomonas aeruginosa* (ATCC27853), *Salmonella* Typhi and *Vibrio cholerae*), a pathogenic yeast (*Candida albicans* (ATCC90028)), and a pathogenic fungus (*Aspergillus fumigatus* AF293). The bacteria were grown on Mueller-Hinton Agar (MHA) at 35°C for 18 h; the yeast was grown on Sabouraud dextrose agar (SDA) at 28°C for 24–48 h; and the filamentous fungus was grown on SDA at 28°C for 48 h. After incubation, inhibition zones were reported as the mean of the well diameter (8 mm) plus the clearing zone in triplicate measurements. Vancomycin and gentamicin were used as standard antibacterial agents and amphotericin B and miconazole were used as standard antifungal agents.

### Diversity and data analysis

The diversity of the fungal isolates associated with *H*. *scabra* was determined by evaluating species richness based on the Menhinick Index (Dmn) [41]. Species diversity was measured by the Shannon (H’) Index [42]. The colonization rate (%CR) was calculated as the total number of tissue segments infected by fungi divided by the total number of segments tested. Relative frequency (%RF) represented the frequency of certain fungal genera divided by the total number of fungal isolates. The statistical analysis was analyzed using Graph Pad Prism, version 7.0 (GraphPad Software, La Jolla, CA).

## Results

### *Holothuria scabra* associated mycobiota

**Fig 2:**
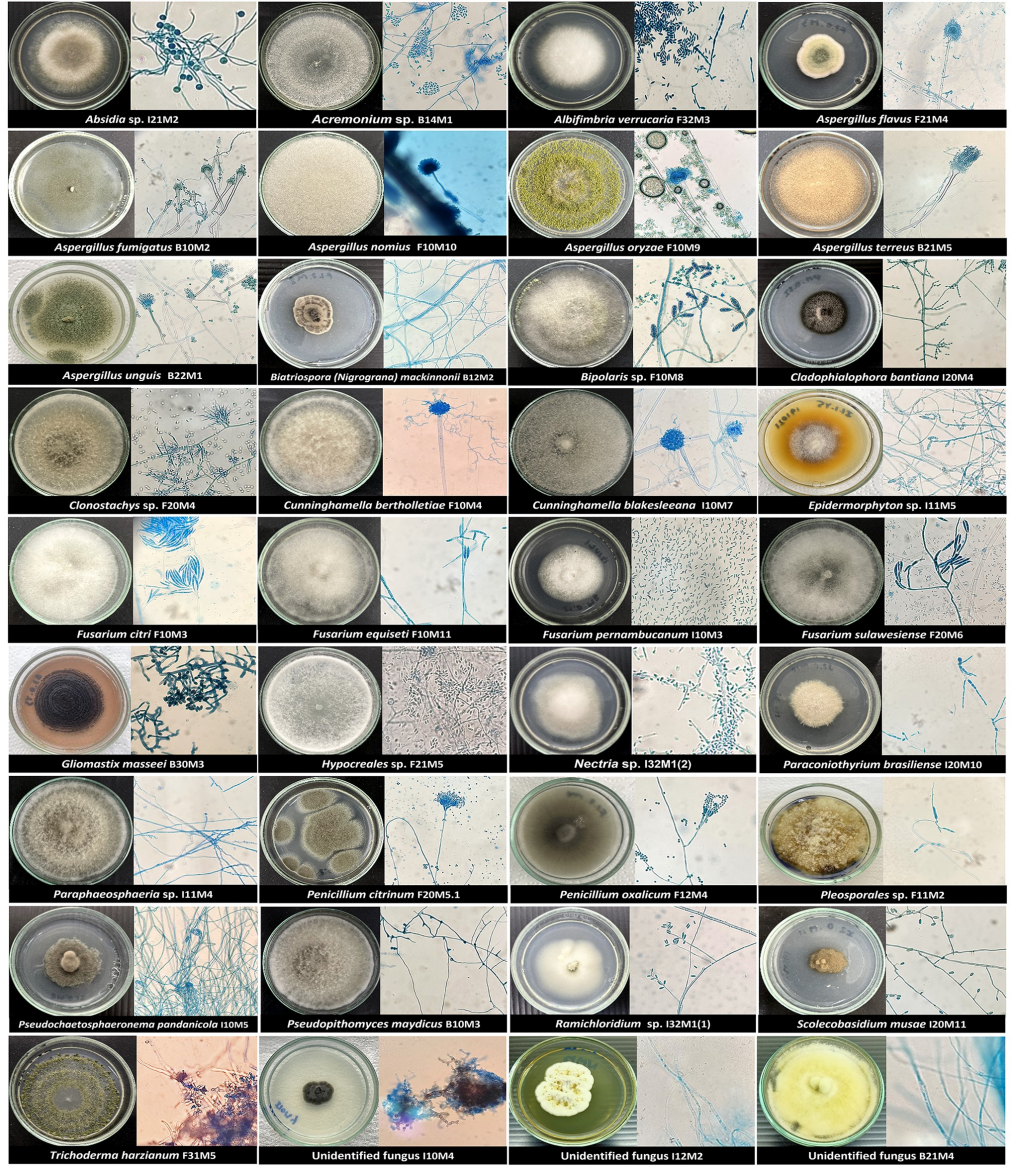
. Fungi isolated from *Holothuria scabra*. Fungi isolated from *Holothuria scabra* were classified into thirty-six fungal morphotypes based on their macroscopic and microscopic morphology (magnification x40). All isolates were grown on CMA plates and incubated at 24°C.

**Fig 3:**
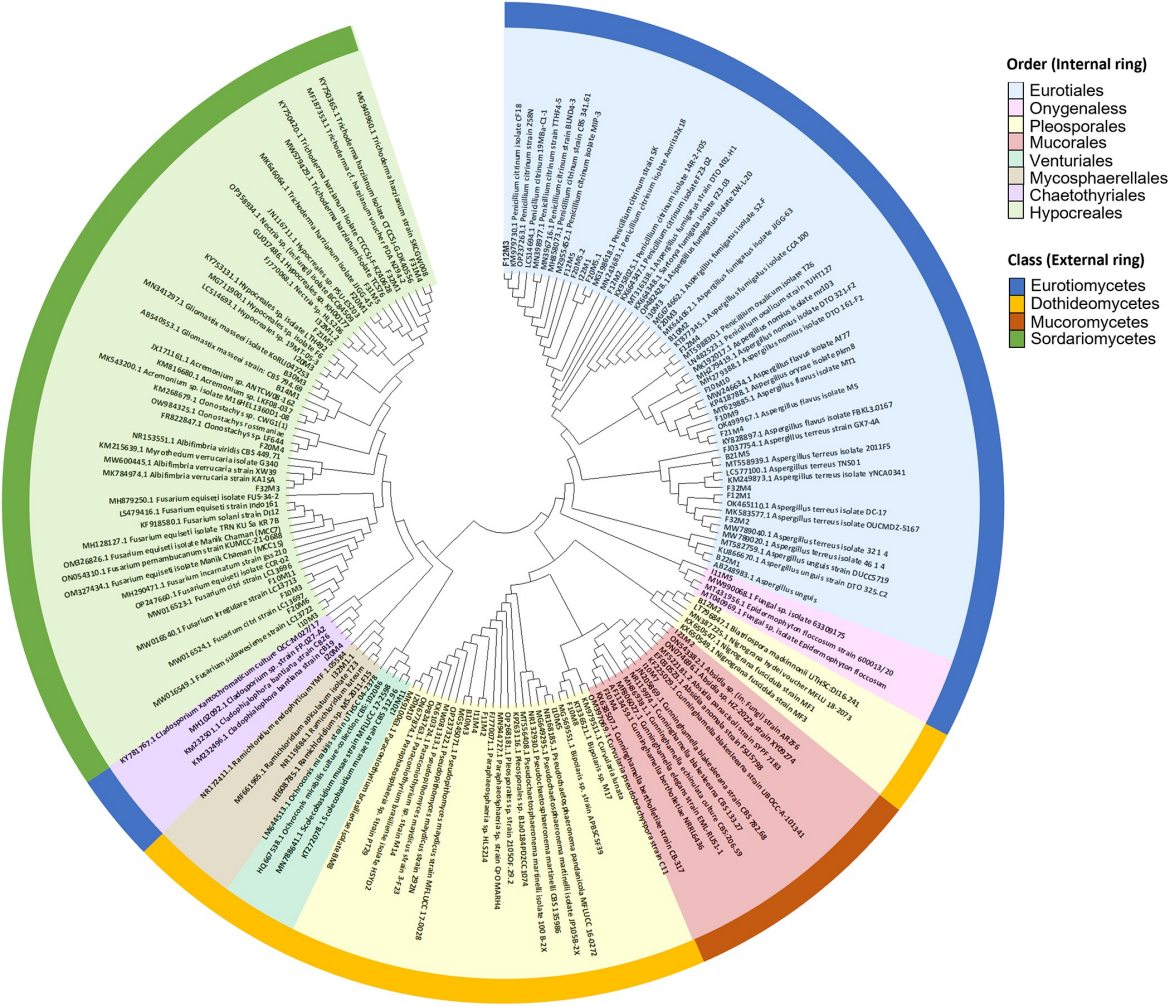
The phylogenetic tree of fungi isolated from *Holothuria scabra* and their allies based on internal transcribed spacer (ITS). The phylogenetic analysis of culturable fungi associated with the *H*. *scabra* produced the above tree generated by the Maximum Likelihood method. The circular phylogenetic tree classifies the isolates at the class, order and genus levels. The inner circle presents orders indicated by different colors, and the outer circle presents classes. Percentages of bootstrap sampling derived from 1000 replications are indicated by the numbers on the tree.

The three sections of six sea cucumbers produced 18 samples, from which 485 fungal isolates were grouped into 50 representative morphotypes. Based on morphological characteristics and molecular identification of the ITS, 33 taxa were obtained plus three unidentified fungal isolates (Fig 2 and Table 1). The phylogenetic tree based on ITS of the isolates at genus, order, and class levels (Fig 3) was generated using the Maximum Likelihood method and Tamura-Nei model, and evolutionary analyses were conducted in MEGA 11 [35]. From the ITS analysis, most species were grouped in their own clade. However, nine taxa, *Absidia* sp., *Acremonium* sp., *Biopolaris* sp., *Clonostachys* sp., *Hypocreales* sp., *Nectria* sp., *Paraphaeosphaeria* sp., *Pleosporales* sp. and *Ramichloridium* sp., could not be identified at the species level despite their best-matched references from the BLASTn search showing similarities higher than 98%. The closest matches of isolates I10M7 and I11M5 were *Cunninghamella blakesleeana* (KF225029.1) and *Epidermophyton* sp. (MT431956.1), respectively. However, their similarities were lower than 96%.

**Table 1:** BLAST analysis based on internal transcribed spacer (ITS) of the marine fungal species recovered from *H*. *scabra* and their closest relatives.

No.*	Code	Closest relative	Accession no.	Similarity (%)
1	I21M2	*Absidia* sp.	ON543382.1	97.74
2	B14M1	*Acremonium* sp.	JX171161.1	98.07
3	F32M3	*Albifimbria verrucaria* strain KA1SA	MK784974.1	99.64
4	F21M4	*Aspergillus flavus* isolate M5	OK499967.1	99.32
5	B10M2	*Aspergillus fumigatus* isolate CCA100	KT877345.1	99.65
	F20M3	*Aspergillus fumigatus* isolate ZW-L-20	OP482428.1	98.81
	I30M3	*Aspergillus fumigatus* isolate S2-F	MG674662.1	99.66
6	F10M10	*Aspergillus nomius* isolate mr103	MK192017.1	98.97
7	F10M9	*Aspergillus oryzae* isolate pkm8	KP418788.1	99.15
8	B21M5	*Aspergillus terreus* isolate 32 1 4	MW789040.1	99.17
	F12M1	*Aspergillus terreus* isolate 46 1 4	MW789020.1	99.50
	F32M2	*Aspergillus terreus* isolate YNCA0341	KM249873.1	99.83
	F32M4	*Aspergillus terreus* TNS01	LC577100.1	99.66
9	B22M1	*Aspergillus unguis* strain DTO 325-C2	KU866670.1	98.77
10	B12M2	*Biatriospora* (*Nigrograna*) *mackinnonii* UTHSC: DI16-241	LT796847.1	99.14
11	F10M8	*Bipolaris* sp.	MG569551.1	98.33
12	I20M4	*Cladophialophora bantiana*	KM232496.1	97.19
13	F20M4	*Clonostachys* sp.	FR822847.1	97.56
14	F10M4	*Cunninghamella bertholletiae*	KX638507.1	99.00
15	I10M7	*Cunninghamella blakesleeana* strain UBOCC-A-101341	KF225029.1	94.39
16	I11M5	*Epidermophyton* sp.	MT431956.1	95.22
17	F10M3	*Fusarium citri* strain LC13697	MW016524.1	99.44
18	F10M11	*Fusarium equiseti* isolate Manik Chaman (MCC15)	OM327434.1	98.73
19	I10M3	*Fusarium pernambucanum* strain KUMCC-21-0688	ON054310.1	99.26
20	F20M6	*Fusarium sulawesiense* strain LC13722	MW016549.1	99.81
21	B30M3	*Gliomastix masseei* isolate KoRLI047253	MN341297.1	98.63
22	F21M5	*Hypocreales* sp. isolate F6	MG711900.1	99.31
	I20M3	*Hypocreales* sp. isolate LTH492	KY753131.1	99.82
23	I32M1(2)	*Nectria* sp. HLS206	FJ770068.1	99.30
24	I20M10	*Paraconiothyrium brasiliense* isolate HSYD2	OP824763.1	99.63
25	I11M4	*Paraphaeosphaeria* sp. strain CPO MARH4	MN944727.1	99.13
26	F12M2	*Penicillium citrinum* strain TTHF4-5	MN398977.1	99.63
	F12M3	*Penicillium citrinum* strain BLND4-3	MN396716.1	99.81
	F12M5	*Penicillium citrinum* 19MBa-C1-1	LC514694.1	98.56
	F20M5.1	*Penicillium citrinum* strain SK	MG198618.1	99.44
	F20M5.2	*Penicillium citrinum* strain TTHF4-5	MN398977.1	99.44
	I22M1	*Penicillium citrinum* isolate MIP-3	MZ955452.1	99.25
27	F12M4	*Penicillium oxalicum*	MT598830.1	99.71
28	F11M2	*Pleosporales* sp. strain 2105OF.29.2	OP268181.1	99.00
29	I10M5	*Pseudochaetosphaeronema pandanicola* MFLUCC 16–0272	NR168185.1	99.61
30	B10M3	*Pseudopithomyces maydicus* strain 3-F23	MW081313.1	98.83
31	I32M1(1)	*Ramichloridium* sp.	HE608785.1	98.07
32	I20M11	*Scolecobasidium musae* strain MFLUCC 17–2598	MN788641.1	97.19
33	F20M1	*Trichoderma* cf. *harzianum* voucher PDA N294-2	MF187353.1	99.19
	F31M4	*Trichoderma harzianum* strain SKCGW008	MG940960.1	100.00
	F31M5	*Trichoderma harzianum* isolate CTCCSJ-G-HB40551	KY750325.1	99.67
	F32M1	*Trichoderma harzianum* isolate JJGG-45	MK646064.1	99.51
34	I10M4	Unidentified fungus	-	-
35	I12M2	Unidentified fungus	-	-
36	B21M4	Unidentified fungus	-	-

*Number of fungal morphotypes

Since the phylogenetic relationships of some fungal isolates were not clear from the ITS analysis, phylogenetic trees based on nuclear large subunit rDNA (LSU) and β-tubulin sequences were constructed to clarify these relationships (S2 Table and S1 Fig). The outlines of LSU-based and β-tubulin-based trees were similar to the ITS-based tree. Based on LSU and β-tubulin sequences, five taxa, *Absidia* sp., *Acremonium* sp., *Clonostachys* sp., *Hypocreales* sp. and *Pleosporales* sp., still could not be identified at the species level even though they were grouped with references in their own clades and the results were supported by the ITS-based identifications. In addition, *Acremonium* sp. and *Hypocreales* sp. did not form clades with their references but instead formed their own clades (S1 Fig). Therefore, their phylogenetic relationships were also correctly represented in the phylogenetic tree based on ITS sequences. The *Cunninghamella* isolate I10M7 had the closest match to *Cunninghamella* sp. (MW699591.1) with a high similarity when analyzed using LSU. This result was not consistent with the ITS-based analysis. Conversely, *Epidermophyton* sp. I11M5 was classified at the species level with high similarity when analyzed using LSU and β-tubulin sequences, and the species was placed in the monophyletic clade of *E*. *floccosum* with a high bootstrap support.

The retrieved isolates were classified into 23 genera, belonging to the two phyla Ascomycota (97.7%) and Mucoromycota (2.3%), four classes Eurotiomycetes (17.4%), Dothideomycetes (39.1%), Mucoromycetes (8.7%) and Sordariomycetes (34.8%), and eight orders Chaetothyriales (4.3%), Eurotiales (8.7%), Hypocreales (34.8%), Mucorales (8.7%), Mycosphaerellales (4.3%), Onygenales (4.3%), Pleosporales (30.4%) and Venturiales (4.3%). The 23 fungal genera were *Absidia*, *Acremonium*, *Albifimbria*, *Aspergillus*, *Biatriospora*, *Bipolaris*, *Cladophialophora*, *Clonostachys*, *Cunninghamella*, *Epidermophyton*, *Fusarium*, *Gliomastix*, *Hypocreales*, *Nectria*, *Paraconiothyrium*, *Paraphaeosphaeria*, *Penicillium*, *Pleosporales*, *Pseudochaetosphaeronema*, *Pseudopithomyces*, *Ramichloridium*, *Scolecobasidium* and *Trichoderma*. *Aspergillus* was the most represented genus (16.7% of the total species) and species belonging to the genus were mainly members of the subgenus *Circumdati* section *Flavi* (*A*. *flavus*, *A*. *nomius* and *A*. *oryzae*), subgenus *Circumdati* section *Terrei* (*A*. *terreus*), subgenus *Fumigati* section *Fumigati* (*A*. *fumigatus*) and subgenus *Nidulantes* section *Nidulantes* (*A*. *unguis*). Other genera representing more than one species were *Fusarium* (four species– 11.1% of the total species) and *Cunninghamella* (two species– 5.6% of the total species).

### Distribution and diversity of fungi associated with *H*. *scabra*

Regarding the three sections under investigation (body wall, intestine and faeces), the numbers of fungal species present in each section were significantly different, and fungal species from the intestine were more morphologically diverse. Twenty-one fungal species were associated with the intestine, 18 with the faeces and 8 with the body wall (Table 2 and S3 Table). Twenty-eight species were isolated from one section only and eight were isolated from more than one section. Mean fungal loads were highest in the faeces (66.15±13.32 CFU per gdw), followed by the intestine (43.02±4.51 CFU per gdw) and the body wall (17.89±9.13 CFU per gdw). The relative frequency of the fungal species was highest in faeces (77.52%RF), followed by the intestine (13.58%RF) and the body wall (8.86%RF).

**Table 2:** Relative frequency (%RF) of marine fungal species recovered from *H*. *scabra*.

Taxa	Relative frequency (%RF)			
	Body wall	Intestine	Faeces	Overall
*Absidia* sp.	-	0.62	-	0.62
*Acremonium* sp.	1.03	0.41	-	1.44
*Albifimbria verrucaria*	0.41	1.03	2.89	4.33
*Aspergillus flavus*	-	-	1.44	1.44
*Aspergillus fumigatus*	1.03	0.82	2.68	4.53
*Aspergillus nomius*	-	-	0.41	0.41
*Aspergillus oryzae*	-	-	2.47	2.47
*Aspergillus terreus*	3.30	-	32.78	36.08
*Aspergillus unguis*	0.41	-	-	0.41
*Biatriospora* (*Nigrograna*) *mackinnonii*	-	0.62	-	0.62
*Bipolaris* sp.	-	-	0.62	0.62
*Cladophialophora bantiana*	-	0.41	1.65	2.06
*Clonostachys* sp.	-	-	0.41	0.41
*Cunninghamella bertholletiae*	-	-	1.24	1.24
*Cunninghamella* sp.	-	0.41	-	0.41
*Epidermophyton floccosum*	-	1.24	-	1.24
*Fusarium citri*	-	-	0.41	0.41
*Fusarium equiseti*	-	-	2.68	2.68
*Fusarium pernambucanum*	-	0.82	-	0.82
*Fusarium sulawesiense*	-	-	0.41	0.41
*Gliomastix masseei*	0.62	-	-	0.62
*Hypocreales* sp.	-	0.41	2.06	2.47
*Nectria* sp.	-	0.82	-	0.82
*Paraconiothyrium brasiliense*	-	0.41	-	0.41
*Paraphaeosphaeria* sp.	-	0.82	-	0.82
*Penicillium citrinum*	-	1.03	19.80	20.83
*Penicillium oxalicum*	0.82	0.62	0.41	1.85
*Pleosporales* sp.	-	-	2.27	2.27
*Pseudochaetosphaeronema pandanicola*	-	1.03	-	1.03
*Pseudopithomyces maydicus*	1.24	-	-	1.24
*Ramichloridium* sp.	-	0.41	-	0.41
*Scolecobasidium musae*	-	0.41	-	0.41
*Trichoderma harzianum*	-	-	2.89	2.89
Unidentified fungus	-	0.41	-	0.41
Unidentified fungus	-	0.62	-	0.62
Unidentified fungus	-	0.21	-	0.21
**Total %RF**	**8.86**	**13.58**	**77.52**	**100.00**
**Number of detected species**	**8**	**21**	**18**	**36**
**Mean fungal load (CFU per gdw)**	**17.89±9.13**	**43.02±4.51**	**66.15±13.32**	**127.06±8.99**

**Fig 4:**
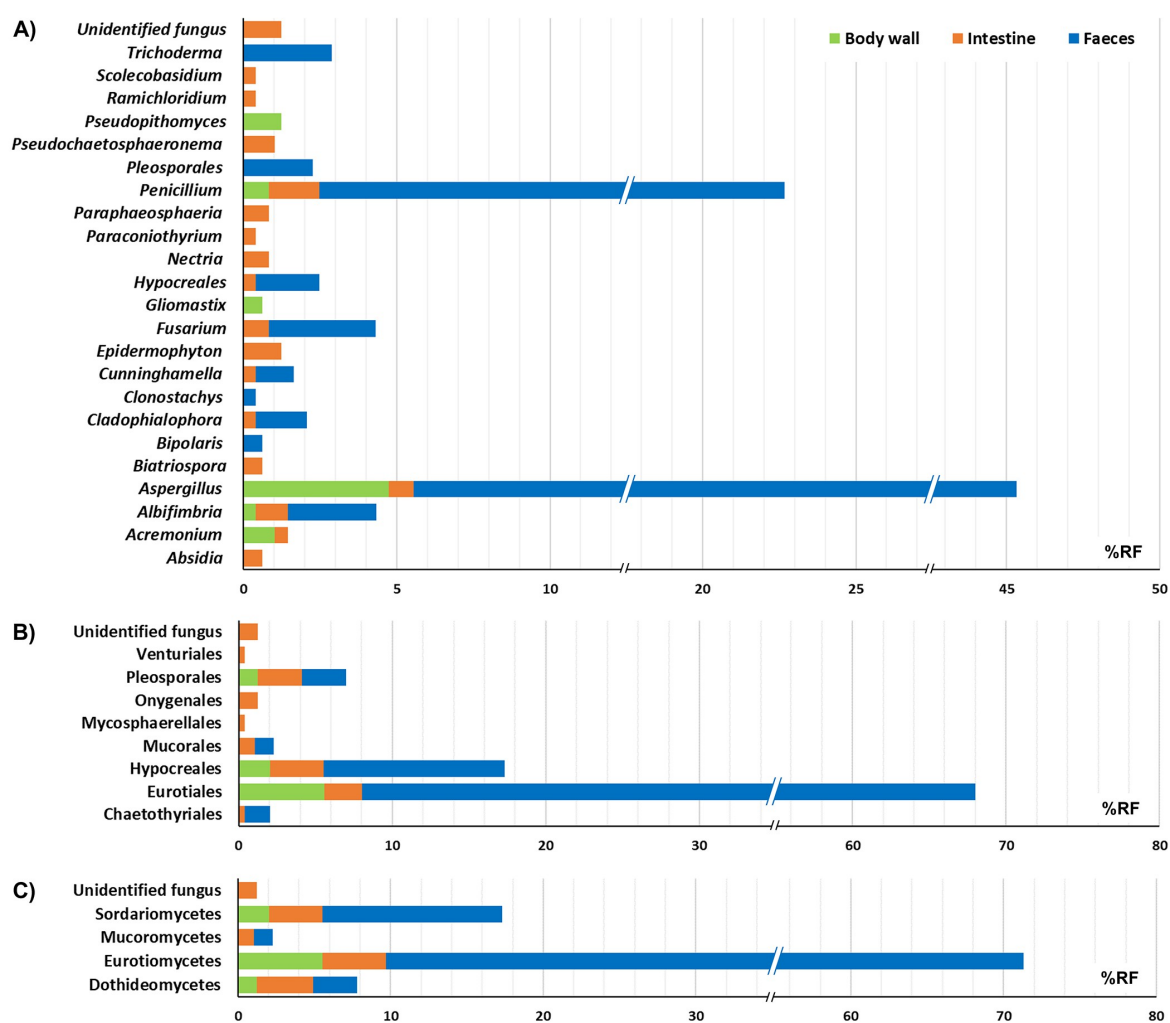
Distribution of fungi associated with *H*. *scabra*. The analysis was based on genus level (A), order level (B), and class level (C).

*Aspergillus* was the dominant genus, detected on the three sections at 45.3%RF (Table 2 and Fig 4A), and was the only ubiquitous genus, with six species (*A*. *flavus*, *A*. *fumigatus*, *A*. *nomius*, *A*. *oryzae*, *A*. *terreus* and *A*. *unguis*) detected on all sections. The %RF of *Aspergillus* was higher in faeces (38.78%RF), followed by body wall (4.74%RF) and intestine (0.82%RF). *A*. *terreus* mainly colonized the faeces (32.78%RF) while *A*. *unguis* was district-specific to the body wall (0.41%RF). *Penicillium* was another section-related dominant genus (22.68%RF), *P*. *citrinum* mainly colonized the faeces (19.80%RF) and *P*. *oxalicum* occurred with a higher %RF in the body wall (0.82) than the faeces (0.41). *A*. *terreus* and *P*. *citrinum* were the species most frequently isolated from faeces; *Epidermophyton* sp., *P*. *citrinum*, *Albifimbria verrucaria* and *Pseudochaetosphaeronema pandanicola* were the species most frequently isolated from intestine content; and *A*. *terreus*, *A*. *fumigatus*, *Acremonium* sp. and *Pseudopithomyces maydicus* were the species most frequently isolated from body wall.

Our results demonstrated that the distribution of fungi associated with *H*. *scabra* varied in different sections of the organism (Fig 4A–4C). Faeces were mainly colonized by the genera *Aspergillus* (39.78%RF) and *Penicillium* (20.21%RF), by the orders Eurotiales (59.99%RF) and Hypocreales (11.75%RF), and by the class Eurotiomycetes (61.64%RF). Intestine contents were mainly colonized by the genera *Penicillium* (1.65%RF) and *Epidermophyton* (1.24%RF), by the orders Hypocreales (3.49%RF), Pleosporales (2.88%RF) and Eurotiales (2.47%RF), and by the classes Eurotiomycetes (4.12%RF) and Sordariomycetes (3.49%RF). The body wall was mainly colonized by the genus *Aspergillus* (4.74%RF), by the orders Eurotiales (5.56%RF), and by the class Eurotiomycetes (5.56%RF).

### Analysis of fungal diversity and species richness

In the analysis of fungal diversity and species richness (Table 3), the Dmn index describes the number of different fungal species represented in an ecological community. With regard to different sea cucumber sections, the Dmn index was highest in the intestine (2.59), followed by the body wall (1.22) and faeces (0.93). Shannon’s index of species diversity (H’) indicated a different biodiversity level among the three sections. The H’ index was highest in the intestine (2.96) but was not significantly different between the body wall and faeces. The highest %CR was obtained from the faeces (%CR, 100), followed by the intestine (%CR, 83.3) and the body wall (%CR, 66.7).

**Table 3:** Colonization (%CR) rates and fungal diversity analysis within districts.

Section	%CR	Dmn	H’
Body wall	66.7	1.22	1.84
Intestine	83.3	2.59	2.96
Faeces	100.0	0.93	1.82
Overall	83.22	1.64	2.36

### Screening for enzymatic activity

**Fig 5:**
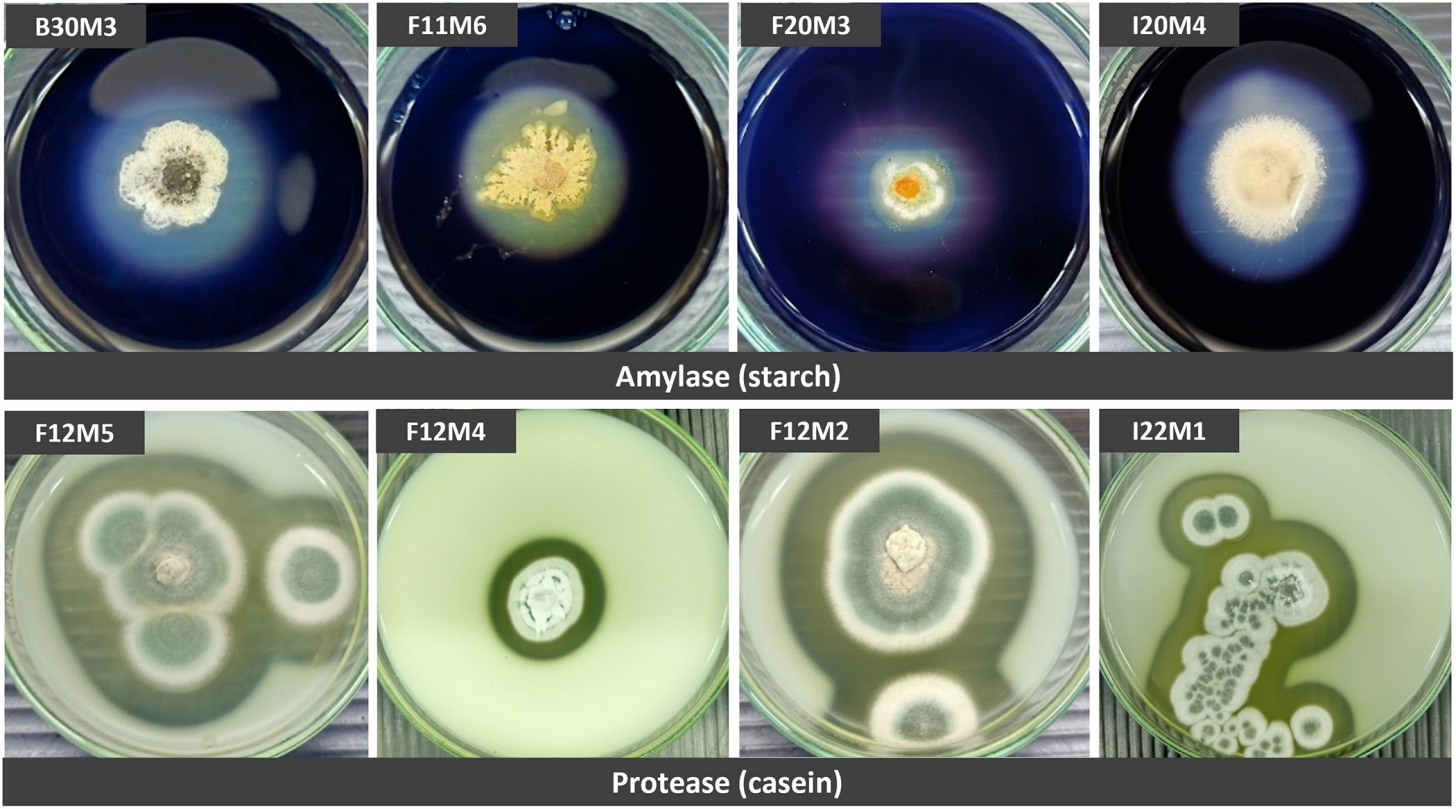
Enzymatic activity test. Eight representative samples of the 16 fungal isolates that exhibited good extracellular enzyme activities.

All the isolates were screened for their ability to produce extracellular proteases, cellulases, amylases and lipases on media containing the respective appropriate substrates. Production of amylases was observed in 511 isolates (Table 4). Positive protease activity was observed in 13 isolates. Thirty-eight out of 54 isolates (30%) did not show any enzymatic activity on any of the substrates, while the production of cellulases and lipases was not detected in any of the isolates. It was observed that most fungi isolated in this study showed moderate enzymatic activity, as indicated by the clear zone diameters around colonies (EA). Most of the isolates which were positive for protease production, showed moderate enzymatic activity; and isolates F11M6, F20M3 and I32M1(2) showed moderate amylase production, producing clear zones of 3.1–6.0 mm (++). High enzymatic activity for amylase production was shown by one isolate, *Gliomastix masseei* B30M3, which showed a clear zone diameter greater than 6 mm (+++). In addition, the EI was used to indicate the ability of each isolate to produce extracellular enzymes. Fungal isolates with an EI equal to or higher than 2 were classified as good candidates for enzyme production [35]. Eight out of 16 isolates exhibited polyenzymatic activity (Table 4 and Fig 5).

**Table 4:** Summary of enzymatic activity of some of the studied fungal isolates. Only isolates positive for at least one substrate are shown.

Strain identity	Isolate code	Enzyme activity (EA)/Enzymatic index (EI)			
		Amylase		Protease	
		EA	EI	EA	EI
**Body wall**					
* Aspergillus unguis*	B22M1	-	-	+	1.3
* Gliomastix masseei*	B30M3	+++	2.3	+	1.3
**Intestine**					
*Hypocreales* sp.	I20M3	-	-	++	1.5
*Nectria* sp.	I32M1(2)	++	1.6	-	-
*Pseudochaetosphaeronema pandanicola*	I10M6	-	-	++	1.6
*Penicillium citrinum*	I22M1	+	1.2	++	1.8
*Ramichloridium* sp.	I32M1(1)	-	-	++	1.5
**Faeces**					
*Aspergillus fumigatus*	F20M3	++	1.5	+	1.3
*Aspergillus terreus*	F10M7	+	1.2	-	-
*Aspergillus terreus*	F11M6	++	1.8	+	1.1
*Aspergillus terreus*	F21M6	+	1.2	-	-
*Aspergillus terreus*	F32M2	+	1.4	+	1.1
*Penicillium citrinum*	F12M2	-	-	++	1.6
*Penicillium citrinum*	F12M5	+	1.2	++	1.7
*Penicillium citrinum*	F20M5	+	1.1	++	1.7
*Penicillium oxalicum*	F12M4	+	1.1	++	1.5

**Key:** Enzyme activity (EA);— = no activity, + = 0–3 mm, ++ = 3.1–6 mm, +++ = >6 mm.

### Screening for dsRNA mycoviruses

**Fig 6:**
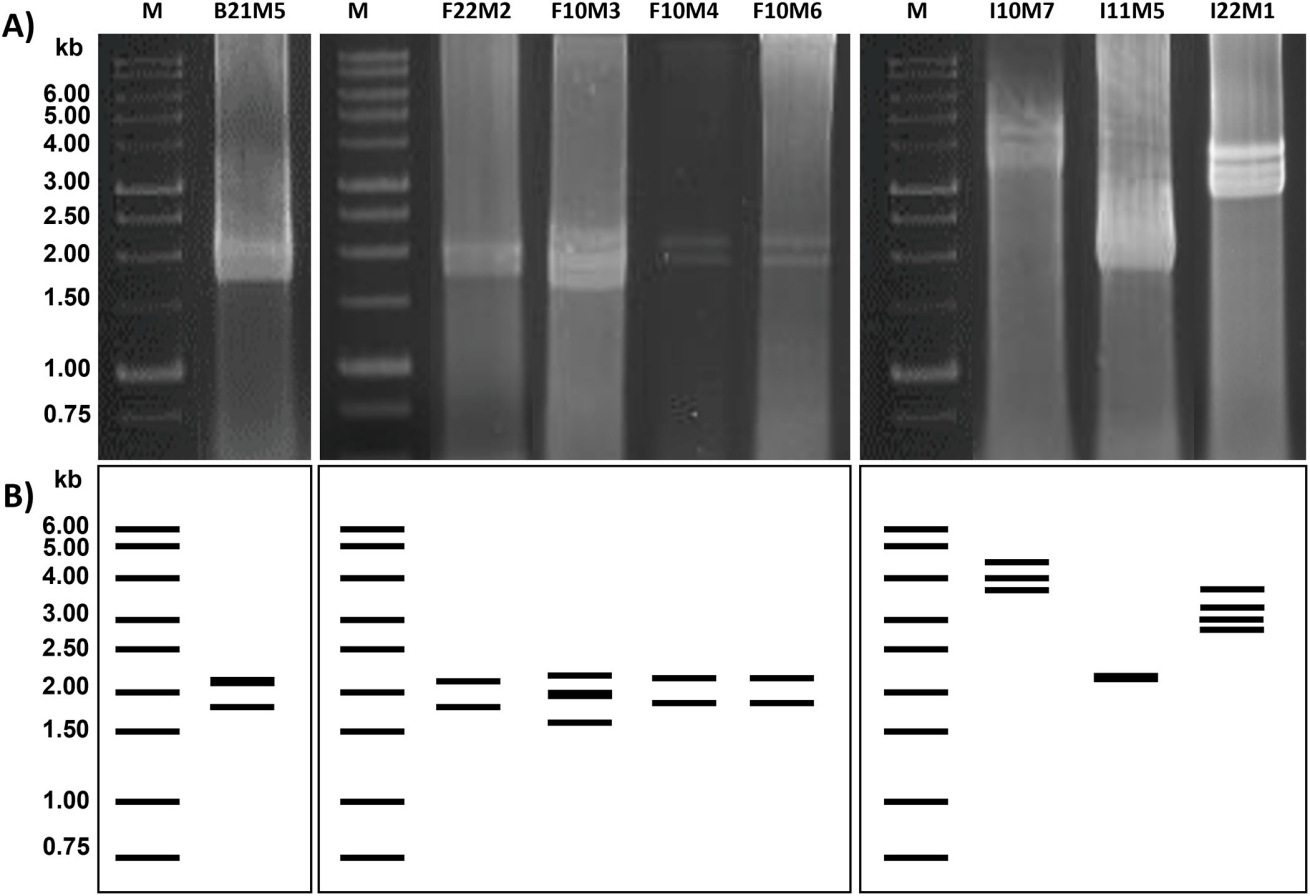
Seven putative dsRNA patterns of the 8 mycovirus-infected strains. A) Gel electrophoresis of isolated dsRNA patterns of fungal isolates. B) The dsRNA profiles displayed in lines.

All 42 fungal isolates were screened for the presence of dsRNA mycoviruses. The dsRNA segments of eight isolates (19%) were detected as bright and distinct bands in gel electrophoresis. Seven different dsRNA patterns were detected, ranging from 1.7 to 4.2 kb in genomic size (Fig 6). Characteristics of each mycovirus were described in Table 5. Four isolates presented two dsRNA fragments of 1.7–2.1 kb and one isolate presented one dsRNA fragment of 2.1 kb. One isolate (*Aspergillus terreus* F10M3) presented three dsRNA fragments of 1.7–2.2 kb and another (*Cunninghamella* sp. I10M7) presented three dsRNA fragments of 3.6–4.2 kb. One isolate (*Penicillium citrinum* I22M1) contained four dsRNA fragments of 2.9–3.9 kb. Enzymatic digestion was performed to confirm dsRNA identities. Primer sets were designed to yield PCR products from the coding regions of the RNA-dependent RNA polymerase (RdRP) and capsid protein (CP) of the partitivirus and chrysovirus previously described by Bhatti *et al*. [43]. However, no amplicon was generated.

**Table 5:** Characteristics of dsRNA mycoviruses in fungi associated with the sea cucumber *H*. *scabra*.

Fungal host	Specimen	Virus code	Segment#	Size (kb)
*Aspergillus terreus* B21M5	Body wall	AsT-B21M5	2	1.7–2.1
*Cunninghamella* sp. I10M7	Intestine	CuBl-I10M7	3	3.6–4.2
*Epidermophyton floccosum* I11M5	Intestine	Ep-I11M5	1	2.1
*Penicillium citrinum* I22M1	Intestine	PeC-I22M1	4	2.9–3.9
*Aspergillus terreus* F22M2	Faeces	AsT-F22M2	2	1.8–2
*Aspergillus terreus* F10M3	Faeces	AsT-F10M3	3	1.7–2.2
*Cunninghamella bertholletiae* F10M4	Faeces	CuBe-F10M4	2	1.8–2.0
*Aspergillus terreus* F10M6	Faeces	AsT-F10M6	2	1.8–2.0

### Antimicrobial assay

**Fig 7:**
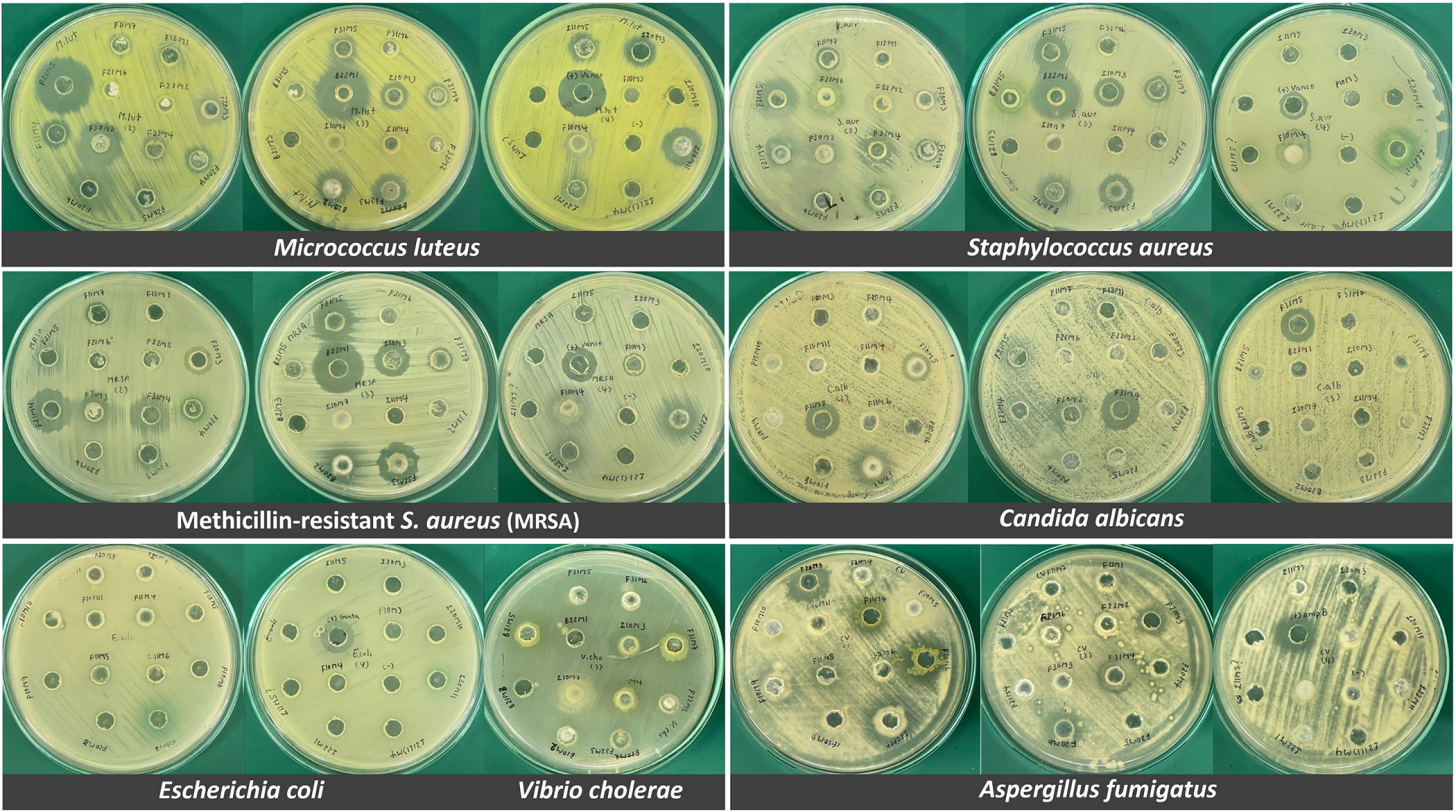
Antimicrobial activity test. The antimicrobial activity of active fungal isolates was determined by the agar well diffusion method against pathogenic bacteria and fungi.

In primary screening, 32 out of 55 isolates (58%) showed antimicrobial activity against at least one pathogen using the culture broth filtrate (Fig 7 and Table 6). Sixteen out of 36 isolates (50%) had positive antimicrobial activity against Gram-positive and Gram-negative bacteria. Meanwhile, seven out of 24 isolates (29%; *Albifimbria verrucaria* F32M3, *Aspergillus flavus* F21M4, *A*. *terreus* F10M7, *Hypocreales* sp. F21M5, *Trichoderma harzianum* F11M5, *T*. *harzianum* F31M4 and *T*. *harzianum* F31M5) had positive antimicrobial activity against Gram-positive and Gram-negative bacteria; and yeast. Three isolates (13%; *Aspergillus terreus* F10M7, *Trichoderma harzianum* F31M4 and *T*. *harzianum* F31M5) showed positive antimicrobial activity against bacteria, yeast and filamentous fungi.

**Table 6:** Screening of antimicrobial activity of fungi isolated from *H*. *scabra* was conducted using the agar well diffusion method.

Isolate	Zone of inhibition (mm)									Total no.
	Pathogenic bacteria							Pathogenic fungi		
	Gram-positive			Gram-negative				Yeast	Mold	
	*ML	SA	MRSA	EC	PA	ST	VC	CA	AF	
**Body wall**										
*Aspergillus unguis* B22M1	**24.7±0.0**	**23.7±0.5**	**23.7±0.0**	**19.3±0.2**	-	-	**22.3±0.0**	-	-	5
**Intestine**										
*Cunninghamella* sp. I10M7	-	-	-	-	-	-	-	-	16.7±2.1	1
*Fusarium pernambucanum* I10M3	14.3±0.2	15.2±0.6	17.1±0.5	-	-	-	18.9±0.0	-	-	4
*Epidermophyton floccosum* I11M5	15.4±0.2	-	-	-	-	-	-	-	-	1
*Hypocreales* sp. I20M3	15.7±1.1	-	-	-	-	-	-	-	-	1
*Penicillium citrinum* I22M1	13.9±0.4	-	**21.8±0.5**	-	-	-	-	-	-	2
*Scolecobasidium musae* I20M11	19.3±0.2	20.3±0.2	17.4±0.6	-	-	-	-	15.7±1.2	-	4
**Faeces**										
*Albifimbria verrucaria* F11M4	-	-	-	-	-	-	**20.6±0.0**	-	**18.4±2.1**	2
*Albifimbria verrucaria* F32M3	17.1±0.5	16.7±0.9	**18.0±0.2**	-	-	-	17.6±0.5	24.5±0.5	-	5
*Aspergillus flavus* F21M4	14.4±0.5	15.6±0.5	**20.7±0.2**	-	-	-	**21.1±1.0**	**30.7±0.0**	-	5
*Aspergillus fumigatus* F20M3	15.0±0.5	13.6±0.4	15.0±0.2	-	-	-	-	-	**18.5±0.5**	4
*Aspergillus nomius* F10M10	-	-	-	-	-	-	**24.0±1.8**	-	-	1
*Aspergillus oryzae* F10M9	**21.0±1.0**	-	-	-	-	14.9±0.6	-	-	**19.6±1.5**	3
*Aspergillus terreus* F10M5	-	15.1±0.0	-	-	-	-	-	18.2±0.0	-	2
*Aspergillus terreus* F10M6	18.4±0.0	**20.3±0.5**	17.4±0.0	-	-	-	-	-	**21.1±0.5**	4
*Aspergillus terreus* F10M7	18.8±0.2	-	-	-	-	-	**27.1±0.5**	15.7±0.3	**24.9±0.7**	4
*Aspergillus terreus* F11M6	-	15.4±1.5	-	-	-	-	19.5±0.4	-	-	2
*Aspergillus terreus* F11M7	-	13.3±0.4	13.2±0.0	-	-	-	-	-	-	2
*Aspergillus terreus* F21M6	8.3±0.3	-	-	14.8±0.5	-	-	**21.0±1.7**	-	-	3
*Aspergillus terreus* F22M2	-	-	-	-	-	-	19.8±0.6	-	-	1
*Aspergillus terreus* F31M7	13.5±0.2	14.7±0.4	14.7±0.2	-	-	-	-	-	-	3
*Clonostachys* sp. F20M4	13.5±0.5	14.4±0.8	13.0±0.6	8.6±0.4	-	-	15.8±0.1	-	14.9±0.4	6
*Cunninghamella bertholletiae* F10M4	14.9±0.0	**18.9±0.0**	**23.3±0.5**	11.8±0.6	-	-	-	-	-	4
*Fusarium citri* F10M3	-	-	14.5±0.1	-	-	-	-	-	-	1
*Fusarium equiseti* F10M11	-	-	-	-	-	-	17.6±0.9	-	-	1
*Fusarium sulawesiense* F20M6	19.6±0.7	-	-	-	-		-	-	18.7±0.5	2
*Hypocreales* sp. F21M5	**26.0±0.0**	18.9±0.0	14.4±0.8	-	-	-	**22.4±0.3**	**14.0±0.4**	-	5
*Penicillium citrinum* F20M5	-	16.0±0.4	13.1±0.5	-	-	-	**19.6±0.9**	-	-	3
*Trichoderma harzianum* F11M5	9.1±0.4	14.9±1.0	15.5±0.5	-	-	-	**19.7+0.5**	**19.5±0.5**	-	5
*Trichoderma harzianum* F31M4	14.2±0.3	13.9±0.6	14.9±0.8	-	-	-	17.6±0.5	**20.0±1.9**	17.2±1.1	6
*Trichoderma harzianum* F31M5	14.3±0.4	17.2±1.1	**20.0±0.4**	**21.9±1.5**	-	**21.4+0.9**	**22.1±0.5**	17.4±1.1	16.6±0.0	8
Unidentified fungus F30M3	19.9+0.9	-	17.8±0.9	-	-	-	16.8±1.0	-	-	3
**Total no.**	22	18	19	4	0	2	18	9	10	
**Positive control**	**ML**	**SA**	**MRSA**	**EC**	**PA**	**ST**	**VC**	**CA**	**AF**	
Vancomycin (20 μg/mL)	23.5±0.0	17.3±0.1	15.3±0.1	28.5±0.1						
Gentamicin (20 μg/mL)					19.7±0.2	17.9±0.7	19.9±0.5			
Amphotericin B (20 μg/mL)								15.2±0.3		
Miconazole (20 μg/mL)									14.3±1.3	

^*^ML: *Micrococcus luteus* (ATCC9341), SA: *Staphylococcus aureus* (ATCC25923), MRSA: methicillin-resistant *S*. *aureus* (MRSA), EC: *Escherichia coli* (ATCC25922), PA: *Pseudomonas aeruginosa* (ATCC27853), ST: *Salmonella* Typhi (ATCC19430), VC: *Vibrio cholerae*, CA: *Candida albicans* (ATCC90028), and AF: *Aspergillus fumigatus* (AF293). Antimicrobial activity that was retained after 4-month storage is bold. The hyphen—indicates no activity.

It was remarkable that *Vibrio cholerae* was inhibited by most of the *Aspergillus* isolates. *Aspergillus unguis* B22M1, which was isolated from body wall tissue, showed strong antibacterial activity against *Staphylococcus aureus*, methicillin-resistant *S*. *aureus* (MRSA) and *Escherichia coli*. *Aspergillus flavus* F21M4 showed the strongest activity against *Candida albicans*, and *Aspergillus terreus* F10M7 showed the strongest activity against *Aspergillus fumigatus*. None of the fungal isolates inhibited *Pseudomonas aeruginosa*. Notably, *Trichoderma harzianum* F31M5 showed the most potent activity against Gram-negative bacteria (*Salmonella* Typhi, *V*. *cholerae* and *E*. *coli*). Results from this study indicated that fungi from sea cucumbers could be a good source of natural antimicrobial products.

The stability of antimicrobial activity was determined after 4-month storage of the fungal cultures in 20% glycerol. Loss of activity against certain test microorganisms was noted for most isolates (S4 Table). Seventeen out of 32 fungal strains (53%) retained their antimicrobial activity to some tested pathogenic bacteria and fungi but showed a decline in antimicrobial activity. However, *Aspergillus unguis* B22M1 and *Hypocreales* sp. F21M5 showed stable antimicrobial activity against *Escherichia coli* and *Candida albicans*, respectively. Therefore, they have a great potential to be used in the development of antimicrobial drugs.

## Discussion

Our study reveals that the Andaman marine echinoderm *Holothuria scabra* is broadly colonized by fungi in its external and internal sections. To date, fungi have been isolated from six species in five genera of sea cucumbers: *Apostichopus japonicus*, *Cucumaria japonica*, *Eupentacta fraudatrix*, *Holothuria nobilis*, *H*. *poli*, and *Stichopus japonicus* (reviewed by [5]). To our knowledge, this is the first report on the fungal community associated with the sea cucumber *H*. *scabra*. The isolated fungal strains belonged to 23 genera. Thirty-three species were from the phyla Ascomycota and Mucoromycota, and three fungal strains were unidentified. Our data showed a significantly higher fungal diversity than has been reported in investigations of other species of sea cucumbers. Marchese et al. [44] described the fungal community associated with the Mediterranean *H*. *poli*, revealing 16 genera and 47 species of fungi. Pivkin [45] and Tan et al. [46] reported fungal communities living in sea cucumbers from the Pacific Ocean, revealing 13 fungal genera from *E*. *fraudatrix*, nine from *A*. *japonicus*, three from *H*. *nobilis* and two from *C*. *japonica*.

Hydrostatic pressure is an important parameter influencing the distribution of microorganisms in the deep sea. The effect of the depth of Holothurian habitat on fungal diversity was described by Pivkin [45]. The deepest sea species (300 m), *C*. *japonica*, had the fewest fungal species while the greatest fungal diversity was associated with *E*. *fraudatrix*, which has an optimal depth of 1.5 m. Similarly, *H*. *poli* and *H*. *scabra*, which inhabit shallow tropical waters, have a high number of fungal species, as reported by Marchese et al. [44] and our study. Twenty-two fungal taxa isolated in this study represent new records for the marine sea cucumber reported worldwide: *Absidia* sp., *Aspergillus unguis*, *Biatriospora* (*Nigrograna*) *mackinnonii*, *Bipolaris* sp., *Cladophialophora bantiana*, *Clonostachys* sp., *Cunninghamella* sp., *C*. *bertholletiae*, *Epidermophyton floccosum*, *Fusarium citri*, *F*. *equiseti*, *F*. *pernambucanum*, *F*. *sulawesiense*, *Gliomastix masseei*, *Hypocreales* sp., *Nectria* sp., *Paraconiothyrium brasiliense*, *Paraphaeosphaeria* sp., *Pseudochaetosphaeronema pandanicola*, *Pseudopithomyces maydicus*, *Ramichloridium* sp. and *Scolecobasidium musae*. This range of species indicates that fungi associated with *H*. *scabra* are diverse. In terms of genus/species recurrence, the Andamanese *H*. *scabra* harboured seven fungal species in common with the Mediterranean *H*. *poli* [44]: *Acremonium*, *Aspergillus*, *Albifimbria* (*Myrothecium*) *verrucaria*, *Penicillium citrinum*, *P*. *oxalicum*, *Pleosporales* and *Trichoderma harzianum*; and three fungal species in common with sea cucumbers from the Pacific Ocean [45]: *A*. *flavus*, *Acremonium* and *Penicillium*.

The high frequency of *Aspergillus* and *Penicillium* found in the fungal community associated with *H*. *scabra* has been found in other sea cucumbers and marine substrates [25, 44, 45, 47, 48]. The presence of recurrent genera suggests a higher adaptation of these taxa to survival in the marine environment and the colonization of sessile echinoderms. The mycobiota isolated from *H*. *scabra* showed similarities with mycobiota isolated from other Andaman substrates: three fungal species also found in brown and red algae samples (*Aspergillus*, *A*. *fumigatus* and *Penicillium*; [47]), five in the tropical seagrass *Enhalus acoroides* (*Aspergillus*, *Bipolaris*, *Hypocreales*, *Penicillium* and *Scolecobasidium*; [25]), five in sponge samples (*Aspergillus*, *A*. *flavus*, *A*. *terreus*, *Penicillium* and *Trichoderma*; [48]), eight in seawater and sediment from mangrove forests (*Biatriospora*, *Acremonium*, *A*. *flavus*, *A*. *fumigatus*, *A*. *nomius*, *Fusarium equiseti*, *Penicillium citrinum* and *P*. *oxalicum*; [47, 49]), and three in marine salterns (*A*. *nomius*, *F*. *equiseti*, and *P*. *oxalicum*; [29]). Notably, some marine fungi are also found in terrestrial environments, indicating the effective adaptive capabilities within the fungal kingdom.

The species composition of fungi isolated from *H*. *scabra* varied in the different substrates sampled. Fungi isolated from the intestine were more diverse, but fungi isolated from faeces were more abundant. The body wall was poorest in both abundance and diversity of fungi. The fungal community associated with the body wall and faeces of *H*. *scabra* comprises unspecific cosmopolitan species that can be found in soils and on various marine substrates, whereas fungi from the internal sections are rather more specific. Since the relationship between fungi and holothurians is inadequately characterized as being either parasitic or symbiotic, Pivkin [45] suggested that fungi associated with the holothurian external body should be considered as epiphytic.

The initial enzymatic assay showed that 16 fungal strains were able to produce extracellular enzymes. Most of the positive isolates presented moderate protease and amylase activity, exhibiting an EI lower than 2 [36]. However, certain isolates could be of interest for industrial applications. The strain that presented the highest EI for amylase production was *Gliomastix masseei* B30M3. Eight out of 16 isolates, mosty *Aspergillus* and *Penicillium*, exhibited polyenzymatic activity. It was evident that the genera *Aspergillus*, *Trichoderma* and *Penicillium* were prominent candidates for amylase and protease production [50]. It has been suggested [51] that damage to holothurian tissues from protease activity is one of the factors that determines the pathogenicity of some fungal strains toward holothurians.

Eight out of 42 fungal strains (19%) harbored dsRNA mycoviruses with different genomic patterns and profiles. Detected viruses contained 1–4 genomic segments ranging in size from 1.7 to 4.2 kb. Most of the fungal strains that contained mycoviruses were *Aspergillus terreus* isolated from the body wall and faeces, which represented 1–2 genome segments of 1.7–2.2 kb. Other fungal strains that contained mycoviruses were *Epidermophyton* I11M5 (1 segment of 2.1 kb), two *Cunninghamella* (2–3 segments of 3.6–4.2 kb) and *Penicillium citrinum* I22M1 (4 segments of 2.9–3.9 kb). To determine whether those mycoviruses were partitiviruses or chrysoviruses, PCR amplification was performed as described by Bhatti et al. [43]. No PCR product was generated, indicating that detected dsRNA mycoviruses either represented members of yet uncharacterized Partitiviridea and Chrysoviridae, or belonged to another virus family. The presence of mycoviruses in fungi associated with other *Holothuria* species has been reported. Among the 48 fungal strains isolated from *H*. *poli* tissues, 10 mycoviruses were identified in eight strains belonging to three fungal genera (*Aspergillus* sp., *Myriodontium* sp. and *Penicillium* sp.), and they belonged to different virus families [8]. An investigation of the mycoviruses associated with sea cucumbers could clarify the effect of mycoviruses on fungal behavior in term of modulating fungal pathogenicity on the host [52].

Antimicrobial activity tests against pathogenic microorganisms showed the ability of many fungal strains associated with *H*. *scabra* to inhibit Gram-positive and Gram-negative bacteria, yeast and filamentous fungi. Our results revealed that members of the *Aspergillus* and *Trichoderma* genera produced the most antimicrobial agents. *Aspergillus terreus* F10M7, *Trichoderma harzianum* F31M4 and *T*. *harzianum* F31M5 showed positive antimicrobial activity against most tested microorganisms, signifying their broad inhibitory effect on common microbial pathogens. These fungal strains exhibited the same antibacterial and antifungal ability as *H*. *scabra* extracts [3]. Notably, *Vibrio cholerae* was inhibited by most of the *H*. *scabra*-associated *Aspergillus* strains in this study. *Aspergillus terreus* from the sea cucumber *A*. *japonicus* was previously reported to produce polyketides, which show diverse bioactive properties that include antibacterial and antifungal activities [53]. *Trichoderma harzianum* F31M5 showed the most potent activity against Gram-negative bacteria (*Salmonella* Typhi, *V*. *cholerae* and *E*. *coli*). Recently, Qi et al. [54] reported the production of the polyketide, Anthraquinone, from sea cucumber-derived *Trichoderma* sp., which showed inhibitory effects against *Pseudomonas putida* and *Vibrio parahaemolyticus*. The ability of marine fungi to synthetize antimicrobial compounds clearly shows their potential in the treatment of microbial infections. Future analyses of antimicrobial activity will involve the identification of the molecules associated with antimicrobial activity and the investigation of chemical profiles produced by positive fungal strains.

## Conclusion

Here, we discovered fungal phylotypes associated with *H*. *scabra* and the selected strains showed enzyme production, antimicrobial potential and mycoviruses. The fungal isolates were classified to 23 genera: *Absidia*, *Acremonium*, *Albifimbria*, *Aspergillus*, *Biatriospora*, *Bipolaris*, *Cladophialophora*, *Clonostachys*, *Cunninghamella*, *Epidermophyton*, *Fusarium*, *Gliomastix*, *Hypocreales*, *Nectria*, *Paraconiothyrium*, *Paraphaeosphaeria*, *Penicillium*, *Pleosporales*, *Pseudochaetosphaeronema*, *Pseudopithomyces*, *Ramichloridium*, *Scolecobasidium* and *Trichoderma*, belonging to the phyla Ascomycota and Mucoromycota, four classes and eight orders. Sixteen fungal strains were positive for protease and amylase activity. Eight fungal stains harbored dsRNA mycoviruses with different genomic patterns and profiles. Thirty-two strains showed antimicrobial activity against pathogenic microorganisms and most were members of the *Aspergillus* and *Trichoderma* genera. Extracellular enzyme and antimicrobial compound production as well as the presence of mycoviruses might play significant roles in interactions of fungi with sea cucumber hosts. To conclude, the investigation of the culturable fungal community associated with *H*. *scabra* is important for the exploration of new fungal strains and to unlock fungal biotechnological potential. We confirmed that fungal strains from sea cucumbers represent a valuable resource for biotechnological applications.

## Supporting information

S1 Fig Phylogenetic tree of the fungi isolated from *Holothuria scabra* and their allies based on nuclear large subunit rDNA (LSU) (A), and β-tubulin (B) sequence alignment. Numbers above branches indicate % bootstrap support. The scale bar indicates nucleotide substitutions per position.
(TIF)

S1 Table Sequences of *Holothuria scabra* fungi. The ITS sequences of marine fungi recovered from *H*. *scabra* deposited in Genbank (accession Nos. OQ835466 to OQ835496).
(DOCX)

S2 Table BLAST analysis based on nuclear large subunit rDNA (LSU) and β-tubulin sequences of the marine fungal species recovered from *H*. *scabra* and their closest relatives. (DOCX)

S3 Table Number of colonies of the representative marine fungal species recovered from *H*. *scabra*. (DOCX)

S4 Table Antimicrobial test. Antimicrobial activity of fungi isolated from *H*. *scabra* after four-month storage.
(DOCX)

S1 Raw image Original gel images. (TIF)
