# Supplementary figures and images for: Diversity and characterization of culturable fungi associated with the marine sea cucumber Holothuria scabra
Source: PLoS One. 2024 Jan 2;19(1):e0296499. doi: 10.1371/journal.pone.0296499 (PMC10760727; doi:10.1371/journal.pone.0296499)

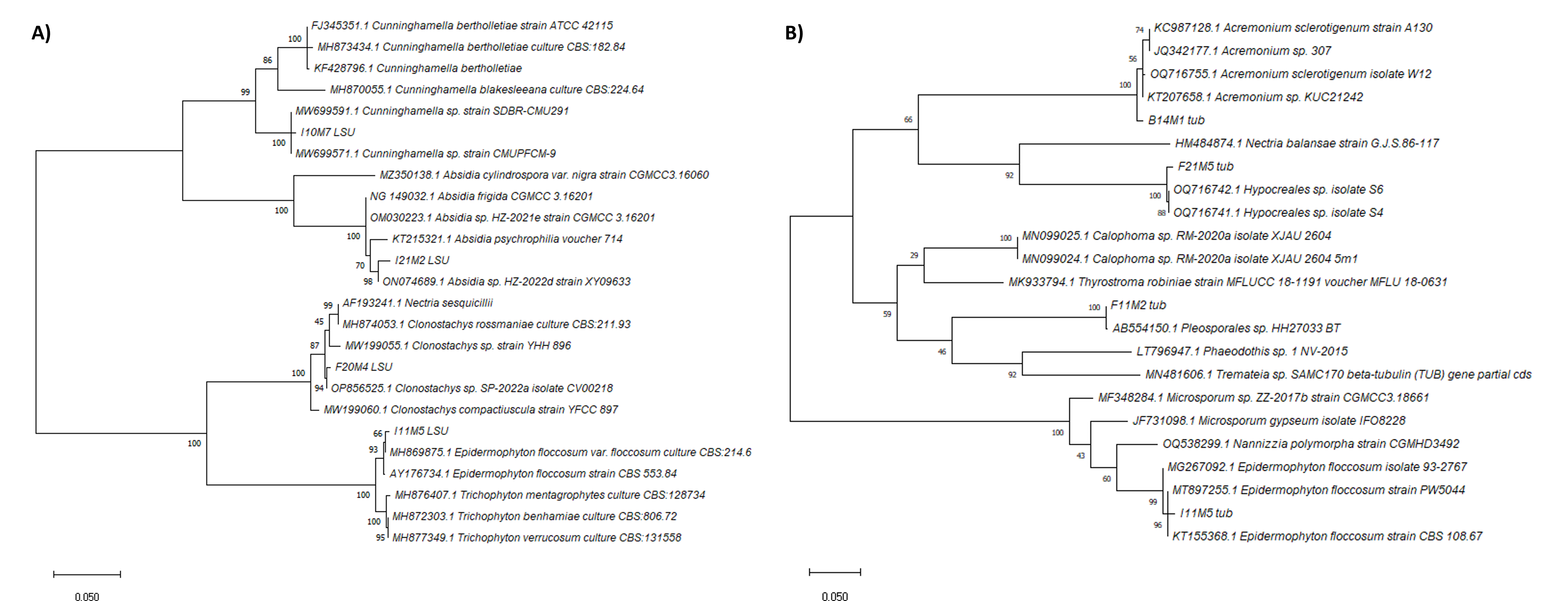

Supplement: S1 Fig — Phylogenetic tree of the fungi isolated from Holothuria scabra and their allies based on nuclear large subunit rDNA (LSU) (A), and β-tubulin (B) sequence alignment. Numbers above branches indicate % bootstrap support. The scale bar indicates nucleotide substitutions per position. (TIF) [file pone.0296499.s001.tif]

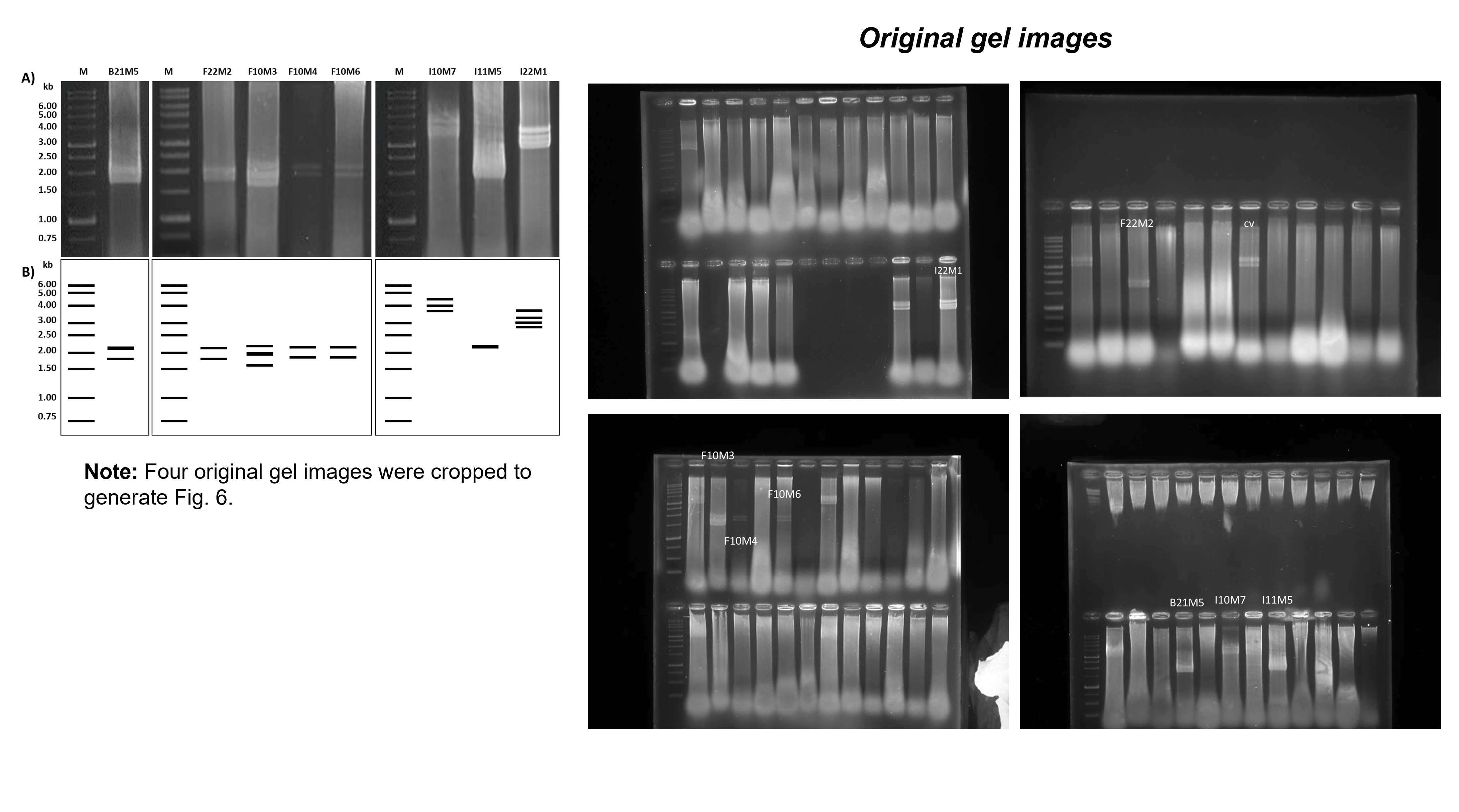

Supplement: S1 Raw image — (TIF) [file pone.0296499.s006.tif]
