# Supplementary material for: Diversity and characterization of culturable fungi associated with the marine sea cucumber Holothuria scabra
Source: PLoS One. 2024 Jan 2;19(1):e0296499. doi: 10.1371/journal.pone.0296499 (PMC10760727; doi:10.1371/journal.pone.0296499)
Supplement: S1 Table — The ITS sequences of marine fungi recovered from H. scabra deposited in Genbank (accession Nos. OQ835466 to OQ835496). (DOCX) [file pone.0296499.s002.docx]

**S1 Table.** **Sequences of *Holothuria scabra*** **fungi.** The ITS sequences of marine fungi recovered from *H. scabra* deposited in Genbank (accession Nos. OQ835466 to OQ835496).

| **Code** | **Taxon** | **Phylum** | **Class** | **Order** | **Accession no.** |
| --- | --- | --- | --- | --- | --- |
| I21M2 | *Absidia* sp. | Mucoromycota | Mucoromycetes | Mucorales |  |
| B14M1 | *Acremonium* sp. | Ascomycota | Sordariomycetes | Hypocreales | OQ835468 |
| F32M3 | *Albifimbria verrucaria* | Ascomycota | Sordariomycetes | Hypocreales |  |
| F21M4 | *Aspergillus flavus* | Ascomycota | Eurotiomycetes | Eurotiales |  |
| B10M2 | *Aspergillus fumigatus* |  |  |  | OQ835466 |
| F20M3 | *Aspergillus fumigatus* |  |  |  | OQ835481 |
| I30M3 | *Aspergillus fumigatus* |  |  |  | OQ835495 |
| F10M10 | *Aspergillus nomius* |  |  |  |  |
| F10M9 | *Aspergillus oryzae* |  |  |  | OQ835473 |
| B21M5 | *Aspergillus terreus* |  |  |  | OQ835469 |
| F12M1 | *Aspergillus terreus* |  |  |  | OQ835476 |
| F32M2 | *Aspergillus terreus* |  |  |  | OQ835488 |
| F32M4 | *Aspergillus terreus* |  |  |  | OQ835489 |
| B22M1 | *Aspergillus unguis* |  |  |  | OQ835470 |
| B12M2 | *Biatriospora* (*Nigrograna*) *mackinnonii* | Ascomycota | Dothideomycetes | Pleosporales |  |
| F10M8 | *Bipolaris* sp. | Ascomycota | Dothideomycetes | Pleosporales | OQ835472 |
| I20M4 | *Cladophialophora* *bantiana* | Ascomycota | Eurotiomycetes | Chaetothyriales | OQ835492 |
| F20M4 | *Clonostachys* sp. | Ascomycota | Sordariomycetes | Hypocreales | OQ835482 |
| F10M4 | *Cunninghamella bertholletiae* | Mucoromycota | Mucoromycetes | Mucorales |  |
| I10M7 | *Cunninghamella blakesleeana* |  |  |  |  |
| I11M5 | *Epidermophyton* sp. | Ascomycota | Eurotiomycetes | Onygenales | OQ835491 |
| F10M3 | *Fusarium citri* | Ascomycota | Sordariomycetes | Hypocreales |  |
| F10M11 | *Fusarium equiseti* |  |  |  | OQ835474 |
| I10M3 | *Fusarium pernambucanum* |  |  |  |  |
| F20M6 | *Fusarium sulawesiense* |  |  |  | OQ835485 |
| B30M3 | *Gliomastix masseei* | Ascomycota | Sordariomycetes | Hypocreales | OQ835471 |
| F21M5 | *Hypocreales* sp. | Ascomycota | Sordariomycetes | Hypocreales |  |
| I20M3 | *Hypocreales* sp. |  |  |  |  |
| I32M1(2) | *Nectria* sp. | Ascomycota | Sordariomycetes | Hypocreales |  |
| I20M10 | *Paraconiothyrium brasiliense* | Ascomycota | Dothideomycetes | Pleosporales |  |
| I11M4 | *Paraphaeosphaeria* sp. | Ascomycota | Dothideomycetes | Pleosporales |  |
| F12M2 | *Penicillium citrinum* | Ascomycota | Eurotiomycetes | Eurotiales | OQ835477 |
| F12M3 | *Penicillium citrinum* |  |  |  |  |
| F12M5 | *Penicillium citrinum* |  |  |  | OQ835479 |
| F20M5.1 | *Penicillium citrinum* |  |  |  | OQ835483 |
| F20M5.2 | *Penicillium citrinum* |  |  |  | OQ835484 |
| I22M1 | *Penicillium citrinum* |  |  |  | OQ835494 |
| F12M4 | *Penicillium oxalicum* |  |  |  | OQ835478 |
| F11M2 | *Pleosporales* sp. | Ascomycota | Dothideomycetes | Pleosporales | OQ835475 |
| I10M5 | *Pseudochaetosphaeronema pandanicola* | Ascomycota | Dothideomycetes | Pleosporales | OQ835490 |
| B10M3 | *Pseudopithomyces maydicus* | Ascomycota | Dothideomycetes | Pleosporales | OQ835467 |
| I32M1(1) | *Ramichloridium* sp. | Ascomycota | Dothideomycetes | Mycosphaerellales | OQ835496 |
| I20M11 | *Scolecobasidium musae* | Ascomycota | Dothideomycetes | Venturiales | OQ835493 |
| F20M1 | *Trichoderma* cf. *harzianum* | Ascomycota | Sordariomycetes | Hypocreales | OQ835480 |
| F31M4 | *Trichoderma harzianum* |  |  |  | OQ835486 |
| F31M5 | *Trichoderma harzianum* |  |  |  |  |
| F32M1 | *Trichoderma harzianum* |  |  |  | OQ835487 |
