# Supplementary material for: Diversity and characterization of culturable fungi associated with the marine sea cucumber Holothuria scabra
Source: PLoS One. 2024 Jan 2;19(1):e0296499. doi: 10.1371/journal.pone.0296499 (PMC10760727; doi:10.1371/journal.pone.0296499)
Supplement: S2 Table — (DOCX) [file pone.0296499.s003.docx]

**S2 Table.** BLAST analysis based on nuclear large subunit rDNA (LSU) and β-tubulin sequences of the marine fungal species recovered from *H. scabra* and their closest relatives.

| **Locus** | **Code** | **Closest relative** | **Accession no.** | **Similarity (%)** |
| --- | --- | --- | --- | --- |
|  |  |  |  |  |
| LSU | I21M2 | *Absidia* sp. HZ-2022d strain XY09633 | ON074689.1 | 98.92 |
|  | F20M4 | *Clonostachys* sp. SP-2022a isolate CV00218 | OP856525.1 | 99.08 |
|  | I10M7 | *Cunninghamella* sp. strain CMUPFCM-9 | MW699571.1 | 99.69 |
|  | I11M5 | *Epidermophyton floccosum var. floccosum* culture CBS:214.63 | MH869875.1 | 99.35 |
| β-tubulin | B14M1 | *Acremonium* sp. KUC21242 | KT207658.1 | 99.06 |
|  | I11M5 | *Epidermophyton floccosum* strain CBS 108.67 | KT155368.1 | 99.01 |
|  | F21M5 | *Hypocreales* sp. isolate S6 | OQ716742.1 | 98.92 |
|  | F11M2 | *Pleosporales* sp. HH27033 BT | AB554150.1 | 99.22 |
