# Supplementary material for: Diversity and characterization of culturable fungi associated with the marine sea cucumber Holothuria scabra
Source: PLoS One. 2024 Jan 2;19(1):e0296499. doi: 10.1371/journal.pone.0296499 (PMC10760727; doi:10.1371/journal.pone.0296499)
Supplement: S3 Table — (DOCX) [file pone.0296499.s004.docx]

**S3 Table.** **Number of colonies of the representative marine fungal species recovered from *H. scabra*.**

| **Code** | **Fungal identity** | **Colony number** | | | |
| --- | --- | --- | --- | --- | --- |
|  |  | **Body wall** | **Intestine** | **Faeces** | **Overall** |
| I21M2 | *Absidia* sp. | - | 3 | - | 3 |
| B14M1 | *Acremonium* sp. | 5 | 2 | - | 7 |
| F32M3 | *Albifimbria verrucaria* | 2 | 5 | 14 | 21 |
| F21M4 | *Aspergillus flavus* | - | - | 7 | 7 |
| B10M2 | *Aspergillus fumigatus* | 5 | 4 | 13 | 22 |
| F20M3 | *Aspergillus fumigatus* |  |  |  |  |
| I30M3 | *Aspergillus fumigatus* |  |  |  |  |
| F10M10 | *Aspergillus nomius* | - | - | 2 | 2 |
| F10M9 | *Aspergillus oryzae* | - | - | 12 | 12 |
| B21M5 | *Aspergillus terreus* | 16 | - | 159 | 175 |
| F12M1 | *Aspergillus terreus* |  |  |  |  |
| F32M2 | *Aspergillus terreus* |  |  |  |  |
| F32M4 | *Aspergillus terreus* |  |  |  |  |
| B22M1 | *Aspergillus unguis* strain | 2 | - | - | 2 |
| B12M2 | *Biatriospora* (*Nigrograna*) *mackinnonii* | - | 3 | - | 3 |
| F10M8 | *Bipolaris* sp. | - | - | 3 | 3 |
| I20M4 | *Cladophialophora* *bantiana* | - | 2 | 8 | 10 |
| F20M4 | *Clonostachys* sp. | - | - | 2 | 2 |
| F10M4 | *Cunninghamella bertholletiae* | - | - | 6 | 6 |
| I10M7 | *Cunninghamella* sp. | - | 2 | - | 2 |
| I11M5 | *Epidermophyton* *floccosum* | - | 6 | - | 6 |
| F10M3 | *Fusarium citri* | - | - | 2 | 2 |
| F10M11 | *Fusarium equiseti* | - | - | 13 | 13 |
| I10M3 | *Fusarium pernambucanum* | - | 4 | - | 4 |
| F20M6 | *Fusarium sulawesiense* | - | - | 2 | 2 |
| B30M3 | *Gliomastix masseei* | 3 | - | - | 3 |
| F21M5 | *Hypocreales* sp. | - | 2 | 10 | 12 |
| I20M3 | *Hypocreales* sp. |  |  |  |  |
| I32M1(2) | *Nectria* sp. | - | 4 | - | 4 |
| I20M10 | *Paraconiothyrium brasiliense* | - | 2 | - | 2 |
| I11M4 | *Paraphaeosphaeria* sp. | - | 4 | - | 4 |
| F12M2 | *Penicillium citrinum* | - | 5 | 96 | 101 |
| F12M3 | *Penicillium citrinum* |  |  |  |  |
| F12M5 | *Penicillium citrinum* |  |  |  |  |
| F20M5.1 | *Penicillium citrinum* |  |  |  |  |
| F20M5.2 | *Penicillium citrinum* |  |  |  |  |
| I22M1 | *Penicillium citrinum* |  |  |  |  |
| F12M4 | *Penicillium oxalicum* | 4 | 3 | 2 | 9 |
| F11M2 | *Pleosporales* sp. | - | - | 11 | 11 |
| I10M5 | *Pseudochaetosphaeronema pandanicola* | - | 5 | - | 5 |
| B10M3 | *Pseudopithomyces maydicus* | 6 | - | - | 6 |
| I32M1(1) | *Ramichloridium* sp. | - | 2 | - | 2 |
| I20M11 | *Scolecobasidium musae* | - | 2 | - | 2 |
| F20M1 | *Trichoderma* cf. *harzianum* | - | - | 14 | 14 |
| F31M4 | *Trichoderma harzianum* |  |  |  |  |
| F31M5 | *Trichoderma harzianum* |  |  |  |  |
| F32M1 | *Trichoderma harzianum* |  |  |  |  |
| I10M4 | Unidentified fungus | - | 2 | - | 2 |
| I12M2 | Unidentified fungus | - | 3 | - | 3 |
| B21M4 | Unidentified fungus | - | 1 | - | 1 |
|  | **Total colony number** | **43** | **66** | **376** | **485** |
