# Supplementary material for: Diversity and characterization of culturable fungi associated with the marine sea cucumber Holothuria scabra
Source: PLoS One. 2024 Jan 2;19(1):e0296499. doi: 10.1371/journal.pone.0296499 (PMC10760727; doi:10.1371/journal.pone.0296499)
Supplement: S4 Table — Antimicrobial activity of fungi isolated from H. scabra after four-month storage. (DOCX) [file pone.0296499.s005.docx]

**S4 Table.** **Antimicrobial test.** Antimicrobial activity of fungi isolated from *H. scabra* after four-month storage.

| **Isolate** | | **Zone of inhibition (mm)** | | | | | | | | | **Total no.** |
| --- | --- | --- | --- | --- | --- | --- | --- | --- | --- | --- | --- |
|  |  | **Pathogenic bacteria** | | | | | | | **Pathogenic fungi** | |  |
|  |  | **Gram-positive** | | | **Gram-negative** | | | | **Yeast** | **Mold** |  |
|  |  | ***ML** | **SA** | **MRSA** | **EC** | **PA** | **ST** | **VC** | **CA** | **AF** |  |
| **Body wall** | | | | | | | | | | | |
|  | *Aspergillus unguis* B22M1 | 20.0+0.4  (24.7+0.0) | 14.2+0.1  (23.7+0.5) | 13.3+0.7  (23.7+0.0) | **18.0+0.1**  (19.3+0.2) | - | - | 14.9+0.0  (22.3+0.0) | - | - | 5 |
| **Intestine** | | | | | | | | | | | |
|  | *Penicillium citrinum* I22M1 | - | - | 13.8+0.9  (21.8+0.5) | - | - | - | - | - | - | 1 |
| **Faeces** | | | | | | | | | | | |
|  | *Albifimbria verrucaria* F11M4 | - | - | - | - | - | - | 12.6+0.2  (20.6+0.0) | - | 10.4+0.2  (18.4+2.1) | 2 |
|  | *Albifimbria verrucaria* F32M3 | - | - | 9.1+0.6  (18.0+0.2) | - | - | - | - | - | - | 1 |
|  | *Aspergillus flavus* F21M4 | - | - | 12.7+0.3  (20.7+0.2) | - | - | - | 13.1+0.2  (21.1+1.0) | 22.7+1.2  (30.7+0.0) | - | 3 |
|  | *Aspergillus fumigatus* F20M3 | - | - | - | - | - | - | - | - | 13.9+1.2  (18.5+0.5) | 1 |
|  | *Aspergillus nomius* F10M10 | - | - | - | - | - | - | 16.0+0.1  (24.0+1.8) | - | - | 1 |
|  | *Aspergillus oryzae* F10M9 | 13.0+0.7  (21.0+1.0) | - | - | - | - | - | - | - | 11.6+1.1  (19.6+1.5) | 2 |
|  | *Aspergillus terreus* F10M6 | - | 12.3+0.3  (20.3+0.5) | - | - | - | - | - | - | 13.1+0.8  (21.1+0.5) | 2 |
|  | *Aspergillus terreus* F10M7 | - | - | - | - | - | - | 19.1+0.7  (27.1+0.5) | - | 16.9+0.5  (24.9+0.7) | 2 |
|  | *Aspergillus terreus* F21M6 | - | - | - | - | - | - | 13.0+0.3  (21.0+1.7) | - | - | 1 |
|  | *Cunninghamella bertholletiae* F10M4 | - | 10.9+0.6  (18.9+0.0) | 15.5+0.2  (23.3+0.5) | - | - | - | - | - | - | 2 |
|  | *Hypocreales* sp. F21M5 | 12.8+0.9  (26.0+0.0) | - | - | - | - | - | 14.4+0.4  (22.4+0.3) | **16.9+0.2**  (14.0+0.4) | - | 3 |
|  | *Penicillium citrinum* F20M5 | - | - | - | - | - | - | 11.6+0.6  (19.6+0.9) | - | - | 1 |
|  | *Trichoderma harzianum* F11M5 | - | - | - | - | - | - | 11.7+0.2  (19.7+0.5) | 11.5+0.9  (19.5+0.5) | - | 2 |
|  | *Trichoderma harzianum* F31M4 | - | - | - | - | - | - | - | 12.0+0.3  (20.0+1.9) | - | 1 |
|  | *Trichoderma harzianum* F31M5 | - | - | 11.9+0.7  (20.0+0.4) | 13.9+0.2  (21.9+1.5) | - | 13.4+0.0  (21.4+0.9) | 14.1+0.5  (22.1+0.5) | - | - | 4 |
| **Total no.** | | 3 | 3 | 6 | 2 | 0 | 1 | 10 | 4 | 5 |  |
| **Positive control** | | **ML** | **SA** | **MRSA** | **EC** | **PA** | **ST** | **VC** | **CA** | **AF** |  |
| Vancomycin (20 µg/mL) | | 23.1±0.7 | 16.9±0.5 | 15.6±0.0 | 28.0±0.6 |  |  |  |  |  |  |
| Gentamicin (20 µg/mL) | |  |  |  |  | 16.6+0.5 | 17.7±0.6 | 19.7±0.8 |  |  |  |
| Amphotericin B (20 µg/mL) | |  |  |  |  |  |  |  | 17.0±0.5 |  |  |
| Miconazole (20 µg/mL) | |  |  |  |  |  |  |  |  | 15.1+0.7 |  |

**^*^**ML: *Micrococcus luteus* (ATCC9341), SA: *Staphylococcus aureus* (ATCC25923), MRSA: methicillin-resistant *S. aureus* (MRSA), EC: *Escherichia coli* (ATCC25922), PA: *Pseudomonas aeruginosa* (ATCC27853), ST: *Salmonella* Typhi (ATCC19430); VC: *Vibrio cholerae*, CA: *Candida* *albicans* (ATCC90028), and AF: *Aspergillus fumigatus* (AF293). Antimicrobial activity that was retained after 4-month storage is bolded. The hyphen - indicates no activity.
